# Superficial parasternal intercostal plane block with ropivacaine versus placebo for opioid exposure after cardiac surgery (EPOCH CardioLink-10): a multicentre, double-blind, randomised trial

**DOI:** 10.1016/j.lana.2026.101508

**Published:** 2026-05-28

**Authors:** Ahmad Alli, C. David Mazer, Fallon Dennis, Hwee Teoh, Kyle Chin, Adrian Quan, S.M. Ali Hassan, Michael Szarek, John D. Tran, Nitish K. Dhingra, Juan P. Ghiringhelli, Fábio de Vasconcelos Papa, Youngseo Lee, Michael J. Ricci, Raj Verma, Kendra L. Derry, Thomas George, Aishwarya Krishnaraj, David A. Hess, Ori D. Rotstein, Richard C. Cook, Ansar Hassan, Terrence M. Yau, Jessica D. Spence, Pawel M. Martinka, Korey Sutherland, Alexander J. Gregory, Christopher D. Noss, Pieter de Jager, James L. Dougherty, Rakesh C. Arora, Subodh Verma

**Affiliations:** a Department of Anesthesia, St Michael’s Hospital–Unity Health Toronto, Toronto, ON, Canada; b Keenan Research Centre for Biomedical Science, Li Ka Shing Knowledge Institute, St Michael’s Hospital–Unity Health Toronto, Toronto, ON, Canada; c Department of Anesthesiology and Pain Medicine, University of Toronto, Toronto, ON, Canada; d Temerty Faculty of Medicine, University of Toronto, Toronto, ON, Canada; e Department of Physiology, University of Toronto, Toronto, ON, Canada; f Department of Pharmacology and Toxicology, University of Toronto, Toronto, ON, Canada; g Division of Cardiac Surgery, St Michael’s Hospital–Unity Health Toronto, Toronto, ON, Canada; h Division of Endocrinology and Metabolism, St Michael’s Hospital–Unity Health Toronto, Toronto, ON, Canada; i Department of Surgery, University of Toronto, Toronto, ON, Canada; j University of Colorado Anschutz Medical Campus and CPC Clinical Research, Aurora, CO, USA; k Mount Sinai Fuster Heart Hospital, Icahn School of Medicine at Mount Sinai, New York, NY, USA; l State University of New York Downstate School of Public Health, Brooklyn, NY, USA; m Division of Cardiovascular Surgery, Peter Munk Cardiac Centre, University Health Network, Toronto, ON, Canada; n School of Medicine, Royal College of Surgeons in Ireland, Dublin, Ireland; o Robarts Research Institute, Schulich School of Medicine and Dentistry, London, ON, Canada; p Division of General Surgery, St Michael’s Hospital–Unity Health Toronto, Toronto, ON, Canada; q Division of Cardiac Surgery, Vancouver General Hospital, Vancouver, BC, Canada; r Division of Cardiovascular Surgery, University of British Columbia, Vancouver, BC, Canada; s MaineHealth Maine Medical Center, Portland, ME, USA; t Departments of Anesthesia, Critical Care, and Health Research Methods, Evidence, and Impact, Population Health Research Institute, McMaster University, Hamilton, ON, Canada; u Department of Anesthesiology, Pharmacology & Therapeutics, University of British Columbia, Vancouver, BC, Canada; v Department of Anesthesiology and Perioperative Medicine, Royal Columbian Hospital, New Westminster, BC, Canada; w Department of Anesthesiology, Perioperative and Pain Medicine, & Libin Cardiovascular Institute, University of Calgary, Calgary, AB, Canada; x Department of Anesthesia, Pain Management & Perioperative Medicine, Dalhousie University, Halifax, NS, Canada; y Departments of Surgery and Anesthesiology, Northwestern University Feinberg School of Medicine, Chicago, IL, USA

**Keywords:** Perioperative pain management, Cardiac surgery, Opioid consumption superficial parasternal intercostal plane block, SPIP block, Median sternotomy, Postoperative pain, Opioid-sparing analgesia, Ropivacaine

## Abstract

**Background:**

Cardiac surgery performed through a midline chest incision (median sternotomy) is associated with substantial postoperative pain and opioid use, the latter of which remains the dominant modality of analgesia. Contemporary international guidelines prioritise opioid-sparing, multimodal strategies; however, robust randomised evidence to support specific opioid-sparing analgesic approaches in cardiac surgery is lacking.

**Methods:**

EPOCH CardioLink-10 was a pan-Canadian, multicentre, randomised, double-blind, placebo-controlled trial in adults undergoing cardiac surgery via median sternotomy. Participants were assigned to receive bilateral superficial parasternal intercostal plane (SPIP) blocks with either 0.2% ropivacaine or placebo (0.9% sodium chloride), delivered via indwelling catheters. Study interventions were delivered at a basal infusion rate (0.1–1 mL/h) after a 20 mL bolus post-insertion. Bolus dosing of 5 mL every 3–4 h was continued for 48 h. In the ropivacaine arm, this resulted in a maximum total ropivacaine dose of 592 mg over the infusion period. The primary outcome was cumulative opioid consumption over 72 h following catheter insertion, expressed in morphine milligram equivalents (MMEs). Secondary outcomes included subjective pain scores, cumulative use of opioid to discharge, rates of delirium, and participant-reported Quality of Recovery-15 score (ClinicalTrials.govNCT06028126).

**Findings:**

Between Aug 16, 2023, and Dec 22, 2025, 650 patients were screened, and 318 included in the modified intention-to-treat population (164 in the ropivacaine group, 154 in the placebo group). Median age was 67 years (IQR 61–72), 72 (23%) participants were female, 178 (56%) were White, and 69 (22%) were South Asian. Over 72 h, ropivacaine reduced opioid consumption compared with placebo (least-squares mean difference −20.7 MMEs [95% CI −39.0 to −2.3]; p = 0.027). Pain scores and other secondary outcomes were similar between groups. There were no serious block-related complications and there was no pre-specified analysis of adverse events.

**Interpretation:**

The findings of this multicentre, randomised trial suggest that bilateral SPIP blockade with ropivacaine may be a pragmatic option to manage sternotomy pain with lower opioid consumption post-cardiac surgery.

**Funding:**

Grants from the Canadian Institutes of Health Research (CIHR) [Principal Applicant: C D Mazer], the Dr. Timothy & Mrs. Linda Tang Anesthesia Research Fund at the University of Calgary, Calgary, AB [A J Gregory and C D Noss], and the CardioLink Research Trial Platform at St. Michael’s Hospital, Toronto, ON. In-kind support from the Department of Anesthesia, Pain Management & Perioperative Medicine, Dalhousie University, Halifax, NS [P de Jager and J L Dougherty].

**Research in context**

**Evidence before this study**

Major surgery is a leading source of opioid exposure worldwide and a key driver of the international opioid crisis. Cardiac surgery ─ performed over 2 million times annually ─ accounts for a substantial proportion of this exposure due to the need to treat pain from a midline sternal incision. We used a combination of predefined terms and Boolean operators to search PubMed, Embase, Web of Science, Google Scholar and the Cochrane Library for articles published from database inception to March 23, 2026. The following were used: “cardiac surgery OR heart surgery”, “sternotomy”, “parasternal block OR superficial parasternal intercostal plane OR SPIP OR superficial parasternal intercostal nerve”, and “randomi∗ OR RCT OR trial”. We included observational studies, randomised controlled trials, and meta-analyses that reported on superficial parasternal intercostal plane blocks, or cardiac surgery by median sternotomy. The snowballing technique was used to identify additional relevant articles. Searches were restricted to those in humans and published in English. Although international guidelines now call for opioid sparing, multimodal analgesia, these recommendations rest on a limited evidentiary foundation. Existing studies are small, heterogeneous, and largely from single centres. Notably, in a meta-analysis of 27 studies that included randomised controlled trials and observational cohorts reporting at least one of the following outcomes: opioid consumption (in morphine milligram equivalents [MMEs]), intensive care unit length of stay, mechanical ventilation duration, pain scores, and mortality, only eight randomised trials were identified. Evidence for continuous superficial parasternal intercostal plane (SPIP) blockade is particularly limited. As a result, there has been little randomised evidence to guide practice in this high-risk population.

**Added value of this study**

EPOCH CardioLink-10 is, to our knowledge, the first adequately powered, multicentre, double blind, placebo-controlled trial to evaluate continuous intermittent bolus SPIP blockade in cardiac surgery. Conducted across four tertiary centres, SPIP blockade with ropivacaine reduced cumulative opioid exposure 72 h post-surgery, while maintaining similar pain control and recovery, with no safety signal.

**Implications of all the available evidence**

These findings address a critical evidence gap at the intersection of cardiac surgery and the international opioid crisis. Continuous SPIP blockade reduces opioid exposure without trade-offs and is readily implementable across centres. In the absence of prior definitive data, this trial provides a clear evidentiary basis for change. SPIP blockade should be incorporated into routine perioperative care for cardiac surgery and embedded within international guideline recommendations. Failure to adopt proven opioid-sparing strategies in this setting risks perpetuating avoidable harm at scale.

## Introduction

Many patients are first exposed to opioids in the perioperative setting, positioning surgery as a major gateway to opioid use. Although opioids remain highly effective for acute pain management, their liberal use carries well-recognised downstream risks and has contributed to an international opioid crisis that has reached epidemic proportions in countries such as Canada, the United States, and Australia.^1^ In the context of this public health emergency, perioperative opioid prescribing represents a modifiable and immediate target for intervention. Yet, in some surgical disciplines, practice continues to rely heavily on opioids in the absence of definitive evidence to support scalable alternatives.

Cardiac surgery, including coronary artery bypass grafting (CABG), valve procedures, and complex aortic operations, remains one of the most invasive forms of major surgery. It is estimated that each year, more than 2 million patients worldwide undergo cardiac surgery via median sternotomy,^2^^,^^3^ an approach that requires midline division of the breastbone. Persistent postoperative pain is common, affecting 7–17% of cardiac patients receiving a sternotomy and up to 30–50% following CABG.^4^ Inadequately controlled pain can lead to pulmonary complications, delayed mobilisation, prolonged hospitalisation, chronic pain syndromes, and impaired functional recovery. Of note, a study from Denmark (N = 29,815 first-time cardiac surgeries)^5^ and a second from the United States (N = 35,817 CABG and valvular surgeries)^6^ reported that approximately 1 in 10 post-cardiac surgery patients develop persistent opioid use. Given that the extent of opioid prescription and opioid use in the short-term after surgery is a strong predictor for persistent opioid use, mitigating short-term opioid exposure may represent a logical approach to relieve the burden of chronic pain syndrome.^5^^,^^6^

Enhanced Recovery After Surgery (ERAS) protocols emphasise standardised, evidence-based pathways to improve outcomes and explicitly prioritise opioid-sparing strategies.^7^, ^8^, ^9^ However, uptake in cardiac surgery has been inconsistent,^10^ largely because the supporting evidence base remains limited, fragmented, and is predominantly derived from small, heterogeneous studies. The administration of up to 500 morphine milligram equivalents (MMEs) in the first 5 hospital days of a cardiac surgical procedure has been reported^11^ and the biggest driver of variability in opioid prescribing seems to be hospital practice, rather than patient factors.^12^ The disconnect between guideline recommendations and high-quality evidence represents a critical barrier to practice change.

Despite broad implementation of multimodal analgesic approaches across other surgical specialties including colorectal, gynaecologic, and orthopaedic surgery, cardiac surgery still remains an outlier, with slow adoption of ERAS-recommended analgesic strategies and continued reliance on opioid-intensive perioperative care.^6^ The first ERAS consensus guidelines for cardiac surgery were published in 2019 and called for multimodal opioid-sparing analgesia while acknowledging the limited cardiac-specific evidence base.^9^

Regional anaesthetic techniques targeting the chest wall have emerged as promising approaches to mitigate opioid exposure after median sternotomy. Systematic reviews and meta-analyses of randomised controlled trials suggest that parasternal intercostal plane blocks produced significant reductions in postoperative pain scores and morphine-equivalent consumption within the first 24 h after surgery, supporting their integration into multimodal analgesia protocols.^13^^,^^14^ Superficial parasternal intercostal plane (SPIP) blockade offers anatomically targeted analgesia delivered in close proximity to the site of surgical trauma.^15^ The promise of SPIP blockade has however been marred by heterogeneity and the lack of robust randomised evidence evaluating efficacy and safety in cardiac surgery has translated to moderate to very low certainty of evidence.

The EPOCH CardioLink-10 trial was specifically designed to address this critical evidence gap with the goal of strengthening the existing fragile data supporting the incorporation of parasternal intercostal plane blocks into multimodal analgesia protocols for cardiac surgery. In brief, this trial evaluated whether continuous bilateral SPIP blockade with ropivacaine could reduce postoperative opioid requirements in adults undergoing cardiac surgery via median sternotomy.

## Methods

### Study design, oversight and participants

EPOCH CardioLink-10 was a double-blind, randomised, placebo-controlled trial conducted at 4 tertiary-level hospitals across 4 Canadian provinces.^16^ The participating sites, site investigators and research coordinators are listed on pp 2 of the Appendix. The Executive and Steering Committees (Appendix pp 3 to 4) provided oversight of this investigator-initiated and investigator-led trial. The protocol (Appendix) was designed by the principal investigators at the primary site. All co-authors contributed to data interpretation and development of the manuscript and its content.

Individuals were eligible if they were aged 18 years and older and were to undergo cardiac surgery via median sternotomy. The following were exclusion criteria: scheduled for redo sternotomy or non-sternotomy approaches; undergoing surgery within 2 h (emergency procedure); clinically unstable; weighing less than 50 kg; having an active systemic bacterial infection; scheduled for surgery for infective endocarditis; pregnant or nursing; chronic opioid use for more than 6 weeks, actively using illicit drugs, long-term opioid exposure or opioid use and/or chronic pain disorder/syndromes; allergic to amide anaesthetic agents or any components of the study intervention ropivacaine; inability to comply with the study protocol; using an investigational drug or device within 7 days of the screening visit. The full set of inclusion and exclusion criteria is listed in the protocol (Appendix Protocol pp 9). Participant sex was self-reported as male or female. Race/ethnicity information was also self-reported.

### Ethics statement

All study activities were conducted in accordance with the Declaration of Helsinki and Good Clinical Practice guidelines. Participants provided written informed consent before entering the trial. The trial protocol was approved by the applicable institutional review boards (Appendix pp 2). Ethical approval for the coordinating site was obtained on Apr 28, 2023 (Reference: 23–038). A data safety monitoring committee (Appendix pp 4) comprising physician experts in cardiac anaesthesiology and cardiac surgery reviewed the safety data after 50% of the trial population had completed participation. The trial was registered with ClinicalTrials.gov (NCT06028126) on Aug 08, 2023.

### Randomisation and blinding

Screening was conducted at a preoperative visit to determine eligibility. After written informed consent was obtained, participants were randomly assigned 1:1 to receive either SPIP blocks with 0.2% ropivacaine or placebo (0.9% sodium chloride) for 48 h following catheter insertion. Randomisation was implemented via the same electronic data capture system used for data collection. The randomisation list was generated with the R version 4.2.2 blockrand package (version 1.5), with stratification by site, sex and surgery type and a block size of 4. The computer-generated sequence randomisation ensured that the study investigators, participant-facing research coordinators/outcome assessors, data collectors, data analysts, participants, and caregivers were masked to the assigned group. Each site had dedicated team members who were unblinded and responsible for preparing and dispensing visually identical ropivacaine and placebo bags, each with ultraviolet-resistant amber covers to maintain masking. Each site was assigned a unique site number; each participant was assigned a unique participant number, and participants were only ever identified by their unique number throughout the trial. Unblinding procedures were available if clinical treatment was needed e.g., cases of suspected local anaesthetic systemic toxicity or allergic reactions.

### Trial procedures

Cardiac surgical and anaesthetic procedures were performed per local standards of care. Sites were chosen based on their experience with, and adherence to, guideline-directed ERAS protocols.^7^ Catheter insertions and bolus deliveries of the assigned intervention were performed after skin closure by anaesthesiologists with specialised regional block training. Anaesthesiologists responsible for catheter insertion and initial dosing were required to demonstrate, during the site initiation visit, competence at conducting the block and placing the catheters in the correct plane. A description of the block procedure has been published with an accompanying video.^15^ Briefly, needle-guided catheters were inserted bilaterally into a sterile field under ultrasound guidance in the T4–T5 intercostal spaces, advanced into the plane, and secured on the skin. The blinded bags of study intervention were set up in infusion pumps, and the assigned study intervention was delivered via dedicated tubing to each study intervention administration catheter (one pump per catheter). After an initial bolus of 20 mL per side, the basal infusion rate was set at 0.1–1 mL/h per side for 48 h, with an additional 5 mL bolus provided every 3–4 h. A maximum of 592 mg of ropivacaine was administered over the 48-h period. This dose falls within the currently accepted safety limits.^17^^,^^18^ The rationale for the dosing schedule was based on previously published literature concerning catheter-based truncal blocks in cardiac surgery patients.^18^^,^^19^ The recommended ceiling dose for ropivacaine in cardiac surgical patients is 0.25 mg/kg/hour^18^ but use of higher doses has been reported.^18^ Our dosing regimen presumed the lowest includable participant body weight (50 kg) which translated to a maximum of 600 mg over 2 days.

Baseline characteristics, surgical details, medications and lengths of stay were captured from in-hospital charts, electronic medical records, or study-specific documents. Clinical outcomes were obtained via direct in-person assessments or interviews conducted by research coordinators, residents and fellows, or nurses using validated tools. Follow-up telephone interviews with the participants were scheduled for 6 weeks and 3 months after the index surgery.

In-person site initiation visits were conducted by study personnel from the primary site. During these visits, the facilities and study equipment were inspected, the protocol was reviewed, and members of the study team received training on data collection, data entry and adverse event reporting procedures and expectations. Data entered into the centralised, web-based electronic data capture system were reviewed by a dedicated team at the primary site on an ongoing basis. Source data verification occurred regularly for select randomised participants at each site. Data checking tools were used to identify data anomalies and detect inconsistencies. Performances of the intervention blocks were recorded on ultrasound, and regional block specialists at the primary site reviewed randomly selected ultrasound images and video clips of catheter placement to ensure adequate technique.

### Outcomes

The primary efficacy endpoint was cumulative postoperative opioid use, measured as MMEs, 72 h post-catheter insertion. This is a well validated measure that is causally related to opioid-related side effects, and was chosen following input from the Steering Committee, a multidisciplinary group of anaesthesiologists, surgeons, intensivists and nurses. The conversion table for opioid doses is provided in Supplemental Table S1. The secondary endpoints were (1) pain score measured, at rest and while coughing (every 12 h using a standardised numeric rating scale) for the 72 h after catheter insertion; (2) cumulative postoperative opioid use from catheter insertion to discharge from hospital; (3) incidence of delirium for the 72 h following catheter insertion (measured using evidence-based delirium assessment tools; Appendix Protocol pp 7); and (4) the participant-reported Quality of Recovery-15 (QoR-15) score, which has been validated for use in cardiac surgical populations, between 24 and 96 h post-surgery. More comprehensive descriptions of the secondary endpoints and details of the exploratory outcomes are available in the study protocol (Appendix Protocol pp 6–7).

### Statistical analysis

It was estimated that approximately 340 individuals would need to be randomised to provide a power of 90% to detect a mean difference of 10 MMEs, assuming a standard deviation (SD) of 25, a 2-sided type 1 error rate of 5% and an attrition rate of 25%. There is ongoing debate regarding the minimal clinically important difference for opioid requirements following cardiothoracic surgeries. The pre-specified mean difference threshold of 10 MMEs was based on previous literature that found reductions in postoperative opioid use of ∼7.5─10 MMEs with chest wall regional anaesthesia techniques in cardiac surgical patients.^20^^,^^21^ Similarly, the assumed SD was determined from previous randomised evidence of regional anaesthesia in cardiac surgical patients, in which the SD of postoperative opioid requirements was between 22 and 27 MMEs.^21^ Further support that a mean difference of 10 MMEs represents a threshold that could be both detectable and clinically relevant comes from a 2025 meta-analysis of opioid-sparing anaesthetic techniques in cardiac surgery that yielded a significant reduction in pooled mean opioid consumption that in turn was associated with reductions in clinical outcomes including ICU length of stay, duration of mechanical ventilation, and persistent opioid use.^22^ No interim analyses were planned or performed. The Statistical Analysis Plan is available in the Appendix.

Efficacy analyses were performed on the modified intention-to-treat (mITT) population that consisted of all randomised participants in whom a block catheter was placed, and a dose of the assigned study intervention was administered. Treatment classification was based on the randomised treatment assignment.

Continuous variables are expressed as means with SDs or medians with interquartile ranges (IQR; quartile 1 to quartile 3). Categorical variables are expressed as counts and percentages. The analysis of the primary efficacy endpoint was by an analysis of covariance (ANCOVA) model assuming heterogeneity in the treatment group variances with fixed effects for treatment group, sex, body mass index (BMI), and intraoperative MMEs administered (<median versus ≥median), yielding least squares means for each treatment group and for the difference between groups, with associated 95% confidence intervals (CIs) and p-values. A sensitivity analysis jointly modelled the primary efficacy endpoint with incomplete dosing of study treatment due to return to the operating room, a rescue nerve block or other reasons. Analyses of secondary efficacy endpoints were by ANCOVA or logistic regression models.

Two-sided p-values less than 0.05 were considered statistically significant, with no adjustment for multiplicity. Analyses were performed in SAS, version 9.4 (SAS Institute) by an independent academic statistician (MS) at CPC Clinical Research, Aurora, Colorado, U.S.A. who had access to the raw data.

### Role of the funding sources

The trial funders had no role in trial design, data collection, data analysis, data interpretation, or writing of the report. The decision to submit and where were at the discretion of the authors.

## Results

**Fig. 1:**
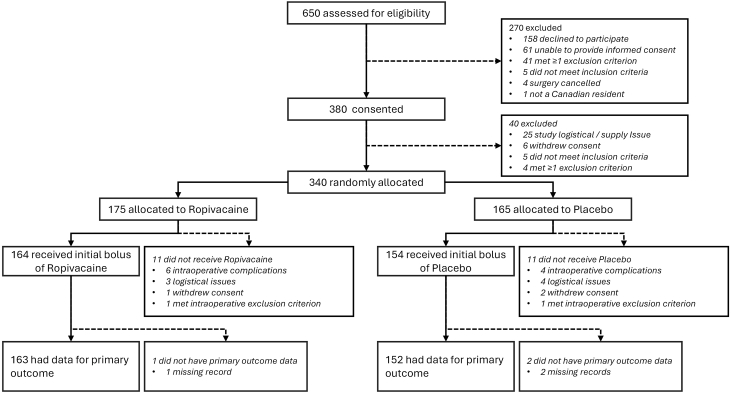
**Trial profile of the EPOCH CardioLink-10 trial**.

Participants were recruited between Aug 16, 2023, and Dec 22, 2025. Among the 650 individuals assessed for eligibility, 380 provided written informed consent, and 340 underwent randomisation (175 [51%] to ropivacaine and 165 [49%] to placebo; Fig. 1). 22 participants ─ 11/175 (6%) from the ropivacaine group and 11/165 (7%) from the placebo group ─ were excluded from the mITT population for reasons summarised in Fig. 1. The incidences of intraoperative exclusions, withdrawal of consent and logistical constraints were not meaningfully different between the treatment groups.

Baseline characteristics of the 318 individuals included in the mITT population are shown in Table 1. Median age was 67 years (IQR 61–72), 72 (23%) participants were female, 178 (56%) were White, and 69 (22%) were South Asian. Most of the 318 participants had coronary artery disease (265, 83%), dyslipidaemia (246, 77%) and hypertension (249, 78%), while 153 (48%) had diabetes and 105 (33%) were living with obesity. Medications on admission were generally balanced across the groups.

**Table 1:** Baseline characteristics of the EPOCH CardioLink-10 participants.

	Ropivacaine	Placebo
	(n = 164^a^)	(n = 154^a^)
Age, years	68 (61–73)	65 (61–70)
Female	42 (25.6)	30 (19.5)
Male	122 (74.4)	124 (80.5)
Race or ethnicity		
White	94 (57.3)	84 (54.5)
South Asian	35 (21.3)	34 (22.1)
Other	31 (18.9)	31 (20.1)
Missing	4 (2.4)	5 (3.2)
BMI, kg/m^2^	27.3 (24.5–31.5)	27.8 (25.0–31.6)
Haemoglobin, g/L	138 (126–148)	138 (127–151)
Creatinine, μmol/L	85 (71–99)	83 (74–97)
Left ventricular ejection fraction, %	55 (51–60)	55 (45–57)
Details of index surgery		
Status		
Elective	133 (81.1)	123 (79.9)
Urgent	26 (15.9)	29 (18.8)
Missing	5 (3.0)	2 (1.3)
Type		
CABG only	116 (70.7)	117 (76.0)
Valvular only	14 (8.5)	22 (14.3)
CABG + Valvular	12 (7.3)	9 (5.8)
Other	22 (13.4)	6 (3.9)
Used internal mammary graft(s) for CABG		
Left internal mammary artery only	126 (76.8)	115 (74.7)
Right internal mammary artery only	0	0
Bilateral internal mammary arteries	3 (1.8)	6 (3.9)
Total surgery time, min	201 (163–237)	195 (162–235)
CPB duration, min	79 (62–102)	76 (62–103)
Intraoperative opioid dosage administered, MMEs	300 (200–400)	300 (200–400)
Cardiovascular history and risk factors		
Coronary artery disease	135 (82.3)	130 (84.4)
Dyslipidaemia	124 (75.6)	122 (79.2)
Hypertension	129 (78.7)	120 (77.9)
Diabetes mellitus	85 (51.8)	68 (44.2)
Smoking (past 6 months)	50 (30.5)	57 (37.0)
Obesity	53 (32.3)	52 (33.8)
Valvular heart disease	50 (30.5)	40 (26.0)
Medications on admission		
Lipid lowering agents	131 (79.9)	130 (84.4)
Antiplatelet agents	117 (71.3)	110 (71.4)
ACEi/ARBs	104 (63.4)	93 (60.4)
β blockers	95 (57.9)	86 (55.8)
Non-insulin antihyperglycaemic agents	71 (43.3)	64 (41.6)
Anti-inflammatory and analgesic agents	46 (28.0)	35 (22.7)
Sedative/neuroleptic agents	25 (15.2)	37 (24.0)
Anticoagulant agents	20 (12.2)	21 (13.6)
Insulin	23 (14.0)	20 (13.0)

Continuous variables are expressed as median (interquartile range). Categorical variables are expressed as n (%). Percentages may not total 100% because of rounding.

Data shown are from the modified intention-to-treat population.

BMI values were calculated based on baseline measurements of bodyweight and height.

ACEi = angiotensin-converting-enzyme inhibitor. ARB = angiotensin receptor blocker. BMI = body mass index. CABG = coronary artery bypass graft. CPB = cardiopulmonary bypass. MMEs = morphine milligram equivalents.

a n vary due to missing values.

Most participants (256/318, 81%) underwent elective surgery, 233/318 (73%) had isolated CABG surgery and 21/318 (7%) had CABG plus valvular surgeries. Amongst those who had CABG surgery, the left internal mammary was used in 241/254 (95%) cases and both internal mammary arteries were used in 9/254 (4%) cases. Median surgery time was 201 min (IQR 163–236) and median cardiopulmonary bypass duration was 79 min (IQR 62–103).

**Fig. 2:**
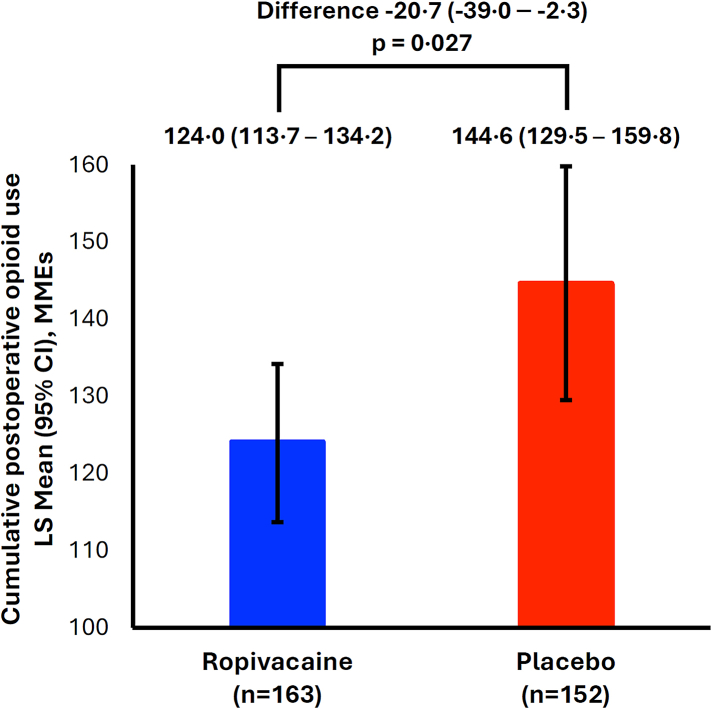
**Primary endpoint of the EPOCH CardioLink-10 trial.** The primary endpoint was cumulative postoperative opioid use, measured as MMEs at 72 h post-catheter insertion. Data are expressed as least squares means and reflect adjustment for sex, body mass index (<median versus ≥median) and intraoperative MMEs administered (<median versus ≥median). CI = confidence interval. LS = least squares. MMEs = morphine milligram equivalents.

**Fig. 3:**
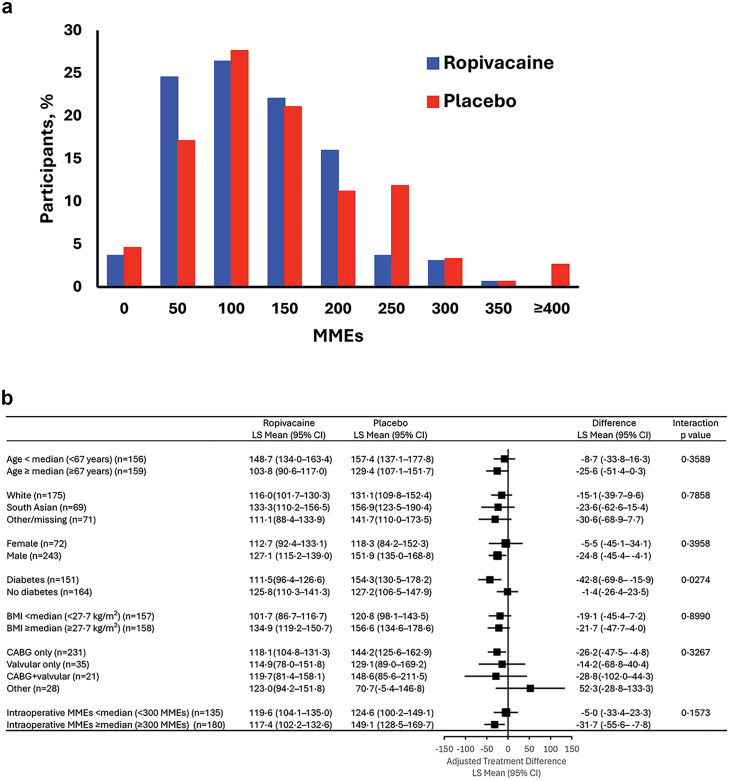
**(A) Distribution of cumulative opioid exposure over the 72 h following catheter insertion and (B) subgroup analyses for opioid exposure.** Forest plot summarising the LS means (95% CIs) of ropivacaine, placebo, and difference between ropivacaine and placebo for subgroups. Results from ANCOVA models with a fixed effects for the treatment group, sex, intraoperative MMEs administered (<median versus ≥median), subgroup (not applicable for sex and intraoperative MMEs subgroup analyses), and the interaction between treatment group and subgroup. BMI = body mass index. CI = confidence interval. LS = least squares. MMEs = morphine milligram equivalents.

The results for the primary outcome are shown in Fig. 2. Mean (SD) cumulative postoperative opioid use was 122.8 MMEs (68.6) for the ropivacaine group and 145.9 MMEs (97.5) for the placebo group. This translated to a significant reduction of 20.7 MMEs with ropivacaine over the first 72 hours post-catheter insertion (p = 0.027). In a sensitivity analysis that jointly accounted for participants who did not receive the full dose of the assigned study intervention (10 in the ropivacaine group, 6 in the placebo group), the reduction in cumulative opioid use with ropivacaine assignment at 72 h remained significant and of similar magnitude (least squares mean difference [95% CI]: −20.7 [−33.6 to −7.7], p = 0.002). The distribution of cumulative opioid exposure in the ropivacaine and placebo groups is shown in Fig. 3A. Opioid exposures for the prespecified subgroups are presented in Fig. 3B. Primary endpoint outcomes for subgroup analyses stratified by age (< and ≥median of 67 years), race/ethnicity (White, South Asian and Other), sex, BMI (< and ≥median of 27.7 kg/m^2^), type of cardiac surgery (CABG only, Valvular, CABG + Valvular and Other) and intraoperative MMEs (< and ≥median of 300 MMEs) remained generally consistent with the overall result.

Results of secondary efficacy outcomes are summarised in Table 2. Consistent with the study hypothesis, patients assigned to ropivacaine required less opioid while maintaining similar pain control. Mean pain scores at rest and with cough over 72 h did not differ between groups (both p > 0.05). Mean (SD) cumulative opioid exposure through to hospital discharge was 174.2 (227.7) MMEs in the ropivacaine group and 192.1 (158.8) MMEs in the placebo group. 22% of the participants experienced an incident of delirium during the first 72 h post-catheter insertion, and the occurrence of delirium was balanced across the groups. QoR-15 scores, collected once between 24 and 96 h following catheter insertion, were available for 82% of the participants and were not different between groups.

**Table 2:** Secondary endpoints of the EPOCH CardioLink-10 trial.

Secondary outcome	Ropivacaine (n = 164)	Placebo (n = 154)	Difference
Pain at Rest^a^ (up to 72 h after catheter insertion)			
Number with ≥1 pain assessment	162	152	
LS Mean (95% CI)	2.84 (2.59–3.08)	2.90 (2.65–3.14)	−0.06 (−0.41 to 0.29)
p value			0.735
Pain with Cough^a^ (up to 72 h after catheter insertion)			
Number with ≥1 pain assessment	162	152	
LS Mean (95% CI)	3.96 (3.66–4.26)	4.08 (3.77–4.39)	−0.12 (−0.56 to 0.32)
p value			0.590
Cumulative postoperative MMEs administered^b^ (through to discharge)			
Number with cumulative MME data	163	152	
LS Mean (95% CI)	176.3 (141.8–210.8)	189.9 (164.5–215.2)	−13.6 (−56.4 to 29.3)
p value			0.534
Quality of recovery-15 score^b^ (assessed once 24–96 h post-surgery)			
Number with QoR-15 data	128	133	
LS Mean (95% CI)	104.0 (100.1–107.8)	105.2 (101.8–108.5)	−1.2 (−6.3 to 3.9)
p value			0.648
**Secondary outcome**	**Ropivacaine (n = 164)**	**Placebo (n = 154)**	**Odds ratio (95% CI)**

Incidence of Delirium^c^^,^^d^ (up to 72 h after catheter insertion)			
n/N (%) with delirium	35/164 (21.3)	36/154 (23.4)	0.84 (0.49, 1.43)
p value			0.520

CI = confidence interval. LS = least squares. MME(s) = morphine milligram equivalent(s). QoR = Quality of Recovery.

a Means, CIs, and p values reflect adjustment for sex and intraoperative MMEs administered (<median versus ≥median).

b Means, CIs, and p values reflect adjustment for sex, body mass index and intraoperative MMEs administered (<median versus ≥median).

c Odds ratio, CIs, and p values reflect adjustment for sex and intraoperative MMEs administered (<median versus ≥median).

d Delirium was determined as defined in the protocol/statistical analysis plan.

The following times from catheter insertion to event are reported as median (quartile 1, quartile 3) for the ropivacaine and placebo groups, respectively: extubation 0.20 days (0.15, 0.30) and 0.19 days (0.15, 0.27), p = 0.474; first opioid analgesia provision 0.27 days (0.21, 0.40) and 0.25 days (0.20, 0.38), p = 0.784; mobilisation (out of bed) 0.70 days (0.48, 0.89) and 0.68 days (0.46, 0.85), p = 0.370. The incidence of nausea/vomiting up to 72 h post-catheter insertion was 68/164 (41.5%) in the ropivacaine group and 50/154 (32.5%) in the placebo group (p = 0.114). Lengths of stay, reported as median (quartile 1, quartile 3) for the ropivacaine and placebo groups, respectively were: intensive care unit 0.98 days (0.86, 1.82) and 0.98 days (0.88, 1.69), p = 0.452; hospital stay 6.3 days (5.2, 8.3) and 6.0 days (5.1, 8.0), p = 0.216. With regards to opioid prescription at discharge, hydromorphone was the second most frequently prescribed analgesic ─ 20.5% of those assigned to ropivacaine and 27.4% of those randomised to placebo received prescriptions for this drug.

Exploratory participant-reported outcomes were collected 6 weeks and 3 months following surgery. PROMIS Pain Interference T-scores^23^ at 6 weeks were similar between groups (least squares difference −0.8 [−3.4 to 1.7]; p = 0.526). They remained comparable between groups (least squares difference 0.8 [−1.1 to 2.8]; p = 0.402) at 3 months. McGill Pain Questionnaire^24^ scores at 3 months were likewise not different between the groups (least squares difference 0.6 [−1.7 to 2.9]; p = 0.604). Taken together, longer-term pain interference and chronic pain burden were similar between treatment groups at both follow-up timepoints.

There were no serious block-related complications. One participant from each group died prior to hospital discharge for reasons unrelated to the study intervention or study block procedure.

## Discussion

EPOCH CardioLink-10 provides robust, multicentre, randomised evidence that a pragmatic, anatomically targeted, and low-cost intervention can decrease perioperative opioid exposure in cardiac surgery. Randomisation to SPIP block with ropivacaine led to a significant decline of 20.7 MMEs over the first 72 hours post-catheter insertion. This reduction in opioid exposure occurred during the critical early postoperative window, when exposure is most strongly linked to persistent downstream opioid use.^25^ Of note, these reductions were achieved without any decrement in analgesia, recovery, or safety, establishing that similar patient-centred outcomes can be attained with substantially less opioid exposure. These findings provide evidence that may inform updates to standard perioperative care in cardiac surgery.

Cardiac surgery remains one of the last major surgical disciplines to rely heavily on opioid-based analgesia, despite mounting recognition of the risks associated with perioperative opioid exposure. Indeed, even in the context of our ERAS-guideline conforming study sites, our placebo group demonstrated a high amount of opioid use that had a similar MME profile to that previously reported.^11^ The present findings demonstrate that targeted regional anaesthesia via SPIP blocks with ropivacaine at a dose that is lower than the maximum recommended limit during the critical early postoperative window reduced opioid requirements during the 72 h following surgery without compromising clinical recovery. Beyond mean opioid exposure, the distribution of cumulative opioid exposure in each group suggests the probability of requiring extremely high opioid doses (≥200 MMEs) can also be reduced with SPIP blocks.

The observed treatment effect is consistent with and expands on the sparse literature evaluating multimodal and regional analgesic strategies in cardiac surgery. Previous studies in the field have suggested reductions in opioid consumption. However, these were small, single-centre trials with heterogeneous methodologies and inconsistent endpoints.^22^^,^^26^ In contrast, a 2025 report evaluating single-shot against continuous SPIP blocks with ropivacaine did not find any difference in opioid consumption up to 48 h post-cardiac surgery.^27^ The investigators randomised 80 participants (1:1) at a single tertiary centre and neither the primary endpoint of numeric rating scale sternal pain score on standardised coughing 24 h following surgery nor the secondary endpoint of early postoperative opioid use (up to 48 h) demonstrated any between-group differences. The approximately 4-fold difference in cohort size, the contrast in study design and distinct interventions likely all contribute at least in part to the divergent results reported for the Jen et al. study and the current work. EPOCH CardioLink-10 provides powered, multicentre, placebo-controlled evidence, thereby addressing a longstanding gap that has limited guideline adoption and standardisation of opioid-sparing approaches in this population. The consistency of the treatment effect across prespecified subgroups further supports the robustness of these findings.

The mechanism underpinning these findings is both biologically plausible and clinically intuitive. SPIP blocks deliver local anaesthetic directly to the site of surgical trauma along the sternotomy, attenuating nociceptive signalling at its source. By reducing afferent pain transmission locally, this approach decreases reliance on systemic opioids while preserving effective analgesia. The comparable pain scores observed between groups reinforce this principle, demonstrating that similar analgesic efficacy can be achieved with lower opioid exposure. This aligns closely with the core tenets of enhanced recovery pathways, that prioritise targeted, multimodal interventions to improve outcomes while minimising treatment-related harm.

Beyond the early postoperative period, the implications of reducing opioid exposure can be considerable. High perioperative opioid doses have been associated with respiratory depression, delirium, gastrointestinal dysfunction, and delayed mobilisation, all of which can prolong hospitalisation and impair recovery. Moreover, early exposure is a key driver of persistent opioid use, which carries risks of long-term dependence, overdose, and increased mortality. Importantly, patients themselves are increasingly aware of and concerned about opioid-related harms, with many expressing a preference for strategies that minimise opioid exposure when effective alternatives are available. Interventions that achieve similar analgesia with lower opioid requirements are therefore not only clinically advantageous but also aligned with evolving patient expectations and shared decision-making.

Postoperative delirium is a common, serious and multifactorial complication amongst cardiac surgery patients with rates typically in the range of 13%–54%.^28^, ^29^, ^30^ Its pathophysiological mechanisms remain poorly elucidated despite several defined risk factors.^28^ In the current work, incidence of delirium 72 h post-catheter insertion was not different between participants assigned to ropivacaine compared to placebo (21.3% versus 23.4%).

Despite the reduction in early postoperative opioid exposure with SPIP blockade, this did not translate into detectable differences in longer-term pain outcomes. This is perhaps unsurprising since chronic post-sternotomy pain is driven by a complex interplay of central sensitisation, neural remodelling, and psychosocial factors that extend well beyond the analgesic window of a 48-h regional block. Furthermore, EPOCH CardioLink-10 was not powered to detect differences in these exploratory endpoints. It is also possible that the background ERAS-based multimodal analgesia provided to both groups attenuated any longer-term divergence between arms.

Postoperative nausea and vomiting affect approximately 42–71% of patients undergoing cardiac surgery^31^ and our cohort reflected this burden, with a peak incidence of 41.5%. Intraoperative opioid use is a well-established risk factor for postoperative nausea and vomiting, and both of our study groups received comparable intraoperative opioid exposure at a median of 300 (IQR 200–400) MMEs. In the current work, the opioid-sparing effect of the intervention was confined to the postoperative period since block placement occurred after skin closure. It is plausible that intraoperative opioid requirements and in turn incidences of postoperative nausea and vomiting may have differed between the groups if the blocks had been placed prior to skin incision.

One of the strengths of the EPOCH CardioLink-10 trial is the use of repeated bolus doses delivered via indwelling catheters, whereas many previous trials used single-shot techniques that have a shorter duration of effect. The trial has several limitations that merit consideration. Female participants represented less than one-quarter (23%) of the study population. While this has direct implications for the generalisability of the current findings to female individuals ─ potentially necessitating a dedicated female only follow-up study ─ it should be noted that this 1:3 female:male distribution aligns with a 2024 representative sample of contemporary cardiac surgical patients in the United States.^32^ The combination of resource restraints with administrative hurdles that delayed site activation and rotating ropivacaine shortages across Canada contributed to asymmetrical enrolment across sites (266 from St. Michael’s Hospital, 46 from Queen Elizabeth II Health Sciences Centre, 16 from Cumming School of Medicine and 12 from Royal Columbian Hospital). We recognise that the observed standard deviation for the primary endpoint is larger than what was used for the sample size estimate and that there is overlap of the standard deviation boundaries. However, the observed mean difference was also larger than anticipated which accordingly allowed for the detection of a significant difference on the primary endpoint. Perioperative prescribing outside the study intervention was left to the discretion of treating clinicians, reflecting real-world practice and thereby improving generalisability of our findings but would have inadvertently introduced some variability in opioid administration despite all sites being ERAS-compliant centres. A modest dosing strategy was selected, so it was not possible for us to ascertain whether higher doses of ropivacaine would have resulted in even greater treatment effects. Our recommendation for guidelines or future studies would be to dose 0.2% ropivacaine by patient body weight rather than non-weight-based dosing volumes. Pain perception remains inherently subjective, and even though validated pain assessment tools were used to mitigate this limitation, visual analogue scales and numerical rating scales do not afford discrete differentiation of sternal, non-sternal, and pre-existing postoperative pain. Multidimensional pain measurement instruments that include functional evaluations may be the way forward for more refined assessments. To the best of our knowledge, EPOCH CardioLink-10 is the largest randomised controlled trial in the cardiac surgery-ERAS domain. However, its sample size of over 300 is relatively small given the annual global cardiac surgery caseload. Even though EPOCH CardioLink-10 demonstrated a reduction in the primary outcome of postoperative opioid exposure, the trial was not powered to detect differences in patient-important clinical outcomes, and the observed reduction in opioid use did not translate into demonstrable improvements in clinical endpoints such as delirium, nausea and vomiting, quality of recovery, or length of stay. Finally, while the intervention is readily implementable, its adoption will require coordination across surgical, anaesthesia, and perioperative care teams.

In conclusion, post-sternotomy pain is multifactorial, necessitating a multimodal analgesic strategy in which SPIP blockade occupies a precise and complementary niche, targeting the parasternal intercostal nerves driving sternotomy pain while avoiding the haematoma risks of neuraxial techniques in anticoagulated patients. Its continuous catheter delivery sustains analgesia across the peak opioid requirement window, and because EPOCH CardioLink-10 was conducted within centres conforming to ERAS guidelines, the observed reduction in opioid exposure represents a benefit above optimised background care. These findings establish SPIP blockade as a viable, safe, pragmatic, and scalable strategy that, in the context of a growing burden of persistent opioid use after cardiac surgery and increasing patient demand for opioid-sparing care, supports the consideration of SPIP blocks as part of routine perioperative pathways and international guideline recommendations. Whether this reduction in opioid exposure ultimately translates into meaningful quality-of-life benefits remains to be determined, but EPOCH CardioLink-10 provides a decisive step in that direction.

## Contributors

SV and CDM chaired the Executive and Steering Committees. AA, CDM and SV conceived the study. AA, CDM, NKD and SV designed the trial protocol. AA, CDM, FD, KC, SMAH, JDT, JPG, FdVP, YL, MJR, RV, KLD, TG, AK, PMM, KS, AJG, CDN, PdJ, JLD and SV conducted the study and collected data. AA, CDM, FD, HT, KC, SMAH and JDT accessed and verified the underlying data. AA, CDM, HT, AQ and SV were responsible for overall trial administration activities. CDM, HT and MS finalised the statistical analysis plan. MS conducted the statistical analysis. AA, CDM, HT, MS, NKD and SV prepared the first draft of the manuscript. AA, CDM, HT and SV incorporated the input of coauthors into subsequent drafts. AA, CDM, FD, HT, KC, AQ, SMAH, MS, JDT, NKD, JPG, FdVP, YL, MJR, RV, KLD, TG, AK, DAH, ODR, RCC, AH, TMY, JDS, PMM, KS, AJG, CDN, PdJ, JLD, RCA and SV were involved in data interpretation, critical revision of the manuscript, approved the final draft, and had final responsibility for the decision to submit the manuscript for publication; they vouch for the accuracy of the data presented and for the fidelity of the study to the protocol. The Executive and Steering Committees (Appendix pp 3 to 4) provided oversight of this investigator-initiated and investigator-led trial.

## Data sharing statement

Data will be made available for academic non-commercial purposes to researchers whose proposed use of the data has been approved, and whose research group includes a qualified statistician or epidemiologist. Data will be provided after completion of a data sharing agreement, ethics review/approval, and protocol registration.

## Declaration of interests

AA reports honoraria for lectures from Edwards Lifesciences.

CDM has received advisory board honoraria/consulting fees from Amgen, Alexion, AstraZeneca, BioAge, Biotest, Boehringer Ingelheim, Cardior, Cytosorbents, Delex, ONA, PhaseBio, Sandoz, Trimedic Therapeutics, and Werfen as well as data safety monitoring board stipends from Beth Israel Deaconess Medical Center, Cerus, Takeda and VarmX.

HT reports receiving personal fees from the Canadian Medical and Surgical Knowledge Translation Research Group and LMC Healthcare.

NKD reports statistical support from Amarin, Boehringer Ingelheim, Eli Lilly, Lexicon Pharmaceuticals, and Sanofi and manuscript publication fee support from Boehringer Ingelheim.

AH reports consulting relationships with AtriCure, Edwards LifeSciences and Intuitive.

TMY reports consulting relationships with Abbott, Aziyo Therapeutics, Bluerock Therapeutics, Gore, HLS Therapeutics Inc., Medtronic, Novo Nordisk and Salutech.

JDS reports receiving consultant fees from VarmX.

AJG is an Executive Board Member of the Enhanced Recovery After Cardiac Surgery Society and sits on the Research Advisory Board of the Canadian Anesthesiologists’ Society.

RCA has received honoraria from Bioporto, Edwards Lifesciences, HLS Therapeutics Inc., Medtronic Inc and Serb Pharmaceuticals, and is on advisory boards for Alexion and Renibus Therapeutics Inc.

SV reports receiving grants and/or research support and/or speaking honoraria from and/or acting as an advisor for Amgen, AstraZeneca, Bayer, Boehringer Ingelheim, Canadian Heart Research Centre, Canadian Medical and Surgical Knowledge Translation Research Group, Eli Lilly, HLS Therapeutics Inc., Humber River Health, Janssen, Merck, Novartis, Novo Nordisk, Pfizer, PhaseBio, S & L Solutions Event Management Inc., Sanofi and Sun Pharma.

All other authors have no relevant conflicts to declare.

## Appendix A Supplementary data

EPOCH_Primary_TLAM_Suppl w CSP, SOC and SAP_SUBMISSION
