# Supplementary material for: Superficial parasternal intercostal plane block with ropivacaine versus placebo for opioid exposure after cardiac surgery (EPOCH CardioLink-10): a multicentre, double-blind, randomised trial
Source: Lancet Reg Health Am. 2026 May 28;60:101508. doi: 10.1016/j.lana.2026.101508 (PMC13235475; doi:10.1016/j.lana.2026.101508)
Supplement: EPOCH_Primary_TLAM_Suppl w CSP, SOC and SAP_SUBMISSION [file mmc1.pdf]

Superficial parasternal intercostal plane block with ropivacaine versus placebo for opioid exposure after cardiac surgery (EPOCH CardioLink-10): a multicentre, double-blind, randomised trial

Ahmad Alli,* C David Mazer,* Fallon Dennis,* Hwee Teoh,* Kyle Chin, Adrian Quan, S M Ali Hassan, Michael Szarek, John D Tran, Nitish K Dhingra, Juan P Ghiringhelli, Fábio de Vasconcelos Papa, Youngseo Lee, Michael J Ricci, Raj Verma, Kendra L Derry, Thomas George, Aishwarya Krishnaraj, David A Hess, Ori D Rotstein, Richard C Cook, Ansar Hassan, Terrence M Yau, Jessica D Spence, Pawel M Martinka, Korey Sutherland, Alexander J Gregory, Christopher D Noss, Pieter de Jager, James L Dougherty, Rakesh C Arora, Subodh Verma

*These authors contributed equally to the work.

ClinicalTrials.gov ID NCT06028126

Appendix Contents

- EPOCH CardioLink-10 Study Sites, Investigators, Research Staff, Research Ethics Approval Details
- Executive Committee
- Steering Committee
- Data and Safety Monitoring Board
- Supplemental Table 1: Milligram morphine equivalent conversions
- Clinical Trial Protocol (Version 30 OCT 2025) plus Summary of Changes from previous protocols
- Statistical Analysis Plan (Version 1.0 / 06FEB2026)

EPOCH CardioLink-10 Study Sites, Investigators, Research Staff, Research Ethics Approval Details

Site Name<br>*(Number Randomized)*	Investigator(s)	Research Coordinator(s)	Ethics Committee	Ethics Reference	Initial Date of Ethics Approval
St Michael's Hospital-Unity Health Toronto, Toronto, ON, Canada<br>*(n= 266)*	PI: Dr Ahmad Alli<br>Co-PIs: Prof Subodh Verma ▪ Prof C David Mazer	Fallon Dennis, BMSc ▪ Kyle Chin, MSc ▪ John D Tran, BSc ▪ Jason Li, BScN ▪ Samson Moses, MBBS	Unity Health Toronto	23-038	April 28, 2023
Queen Elizabeth II Health Sciences Centre, Halifax, NS, Canada<br>*(n= 46)*	PI: Dr James L Dougherty<br>Co-PI: Dr Pieter de Jager	Ashley N Ross Zahavich, MScK, BScK, BScN-RN ▪ Gabrielle M Hanson, MSc, BSc	Nova Scotia Health Research Ethics Board	1030782	August 19, 2024
Cumming School of Medicine, University of Calgary, Calgary, AB, Canada<br>*(n= 16)*	PI: Dr Christopher D Noss<br>Co-I: Dr Alexander J Gregory	Ish Bains, MSc, PhD	Conjoint Health Research Ethics Board	23-1764	May 27, 2024
Royal Columbian Hospital, New Westminster, BC, Canada<br>*(n= 12)*	PI: Dr Pawel Martinka<br>Co-I: Korey Sutherland, MN	Michelle R Mozel, MSc ▪ Jenna Van Roekel, BSc	Fraser Health Research Ethics Board	2025014	March 04, 2025

Co-I, Co-Investigator; PI, Principal Investigator.

Executive Committee

- **Prof Subodh Verma** | St Michael's Hospital-Unity Health Toronto, University of Toronto, Toronto, ON, Canada *(Executive Committee Co-Chair)*
- **Prof C David Mazer** | St Michael's Hospital-Unity Health Toronto, University of Toronto, Toronto, ON, Canada *(Executive Committee Co-Chair)*
- **Dr Ahmad Alli** | St Michael's Hospital-Unity Health Toronto, University of Toronto, Toronto, ON, Canada
- **Adrian Quan, MPhil** | St Michael's Hospital-Unity Health Toronto, Toronto, ON, Canada
- **Hwee Teoh, PhD** | St Michael's Hospital-Unity Health Toronto, Toronto, ON, Canada

Steering Committee

- **Prof Subodh Verma** | St Michael's Hospital-Unity Health Toronto, University of Toronto, Toronto, ON, Canada *(Steering Committee Co-Chair)*
- **Prof C David Mazer** | St Michael's Hospital-Unity Health Toronto, University of Toronto, Toronto, ON, Canada *(Steering Committee Co-Chair)*
- **Dr Corey Adams** | Cumming School of Medicine, University of Calgary, Calgary, AB, Canada
- **Dr Ahmad Alli** | St Michael's Hospital-Unity Health Toronto, University of Toronto, Toronto, ON, Canada
- **Prof Rakesh C Arora** | Northwestern University Feinberg School of Medicine, Chicago, IL, United States
- **Prof Richard C Cook** | Vancouver General Hospital, University of British Columbia, Vancouver, BC, Canada
- **Dr Pieter de Jager** | Queen Elizabeth II Health Sciences Centre, Halifax, NS, Canada
- **Fallon Dennis, BMSc** | St Michael's Hospital-Unity Health Toronto, University of Toronto, Toronto, ON, Canada
- **Dr James L Dougherty** | Queen Elizabeth II Health Sciences Centre, Halifax, NS, Canada
- **Dr Alexander J Gregory** | Cumming School of Medicine, University of Calgary, Calgary, AB, Canada
- **Dr Ansar Hassan** | MaineHealth Maine Medical Center Portland, Portland, ME, United States
- **Dr S M Ali Hassan** | St Michael's Hospital-Unity Health Toronto, University of Toronto, Toronto, ON, Canada
- **Dr Pawel M Martinka** | Royal Columbian Hospital, University of British Columbia, Vancouver, BC, Canada
- **Dr Amine Mazine** | Royal Children's Hospital, Melbourne, VIC, Australia
- **Dr A Dave Nagpal** | London Health Science Centre, Western University, London, ON, Canada

- **Dr Christopher D Noss** | Cumming School of Medicine, University of Calgary, Calgary, AB, Canada
- **Adrian Quan, MPhil** | St Michael's Hospital-Unity Health Toronto, Toronto, ON, Canada
- **Dr Michael J Ricci** | St Michael's Hospital-Unity Health Toronto, University of Toronto, Toronto, ON, Canada
- **Prof Ori D Rotstein** | St Michael's Hospital-Unity Health Toronto, University of Toronto, Toronto, ON, Canada
- **Dr Jessica D Spence** | Population Health Research Institute, McMaster University, Hamilton, ON, Canada
- **Korey Sutherland, MN** | Royal Columbian Hospital, New Westminster, BC, Canada
- **Hwee Teoh, PhD** | St Michael's Hospital-Unity Health Toronto, Toronto, ON, Canada
- **Raj Verma** | Royal College of Surgeons in Ireland, Dublin, Ireland
- **Prof Terrence M Yau** | University Health Network, University of Toronto, Toronto, ON, Canada

Data and Safety Monitoring Board

- **Dr Tarit Saha *(Chair)*** | Kingston General Hospital, Queens University, Kingston, ON, Canada
- **Dr Robert S Kramer** | MaineHealth Maine Medical Center Portland, Tufts University School of Medicine, Portland, ME, United States
- **Prof Étienne De Médicis** | CHUS - Hôpital Fleurimont, Université de Sherbrooke, Sherbrooke, QC, Canada

Supplemental table 1: Morphine milligram equivalent conversions.

**Opioid**	**Administrative Route**	**MME Conversion Factor**
Codeine	Oral	0·15
Fentanyl	Intravenous	0·2
Hydromorphone	Intramuscular	15
Hydromorphone	Intravenous	15
Hydromorphone	Oral	4
Hydromorphone	Subcutaneous	15
Meperidine	Intravenous	0·4
Morphine	Intravenous	3
Morphine	Oral	1
Morphine	Subcutaneous	3
Oxycodone	Oral	1·5
Sufentanil	Intravenous	2
Tramadol	Oral	0·1

MME=morphine milligram equivalent.

EPOCH CardioLink-10 Clinical Trial Protocol (Version 30 OCT 2025) ***plus*** **Summary of Changes from Previous Protocols**

Efficacy of Superficial Parasternal Intercostal Plane Block on Postoperative Pain Control in Patients Undergoing Cardiac Surgery with Median Sternotomy

EPOCH CardioLink-10 Randomized Clinical Trial

Clinical Trial Protocol

1. Protocol Synopsis

Full Title	Efficacy of Superficial Parasternal Intercostal Plane Block on Postoperative Pain Control in Patients Undergoing Cardiac Surgery with Median Sternotomy
Short Title	EPOCH CardioLink-10 RCT
Funding	CardioLink Clinical Trial Platform
Study Population	Adult patients undergoing cardiac surgery via median sternotomy
Study Design	Randomized, double-blinded, parallel-group, multicentre trial of superficial parasternal intercostal plane block with ropivacaine via ultrasound-guided catheter placement versus sham block with saline in cardiac surgical patients undergoing median sternotomy.
Sample Size	340 patients
Study Duration	1-2 years
Study Intervention	Subjects will be randomized (1:1) to receive superficial parasternal intercostal plane block with ropivacaine via ultrasound-guided catheter placement or sham block with saline. Catheters will be inserted into the superficial parasternal intercostal plane bilaterally under ultrasound guidance after skin closure. Following initial bolus dosing at the time of catheter placement, a continuous infusion with intermittent boluses every 3-4 hours of 0.2% ropivacaine, or equivalent volume of 0.9% normal saline, will be delivered via catheters for 48 hours post-catheter insertion.
Duration of Treatment	Treatment will be delivered for 48 hours post-catheter insertion.
Outcomes	• Primary outcome: Cumulative postoperative opioid use measured as Milligram Morphine Equivalent (MME) up to 72 hours following catheter insertion<br>• Secondary outcomes:<br>o Median pain score as measured by a standardized numeric rating scale over 72 hours following catheter insertion (measured at rest and with coughing q12hours ± 2 hours post-catheter insertion)<br>o Cumulative postoperative opioid use measured as Milligram Morphine Equivalent (MME) from catheter insertion until discharge from hospital<br>o Delirium assessed BID for 72 hours following catheter insertion<br>o Quality of Recovery-15 Scale administered 24-96 hours post-surgery.<br>• Tertiary (exploratory) outcomes:<br>o Time from catheter insertion to extubation<br>o Time from catheter insertion to first opioid analgesic provision following extubation<br>o Time from catheter insertion to mobilization<br>o ICU and hospital LOS<br>o Postoperative nausea and vomiting, measured via a 10-point visual analogue scale BID and antiemetic medication requirements up to 72 hours following catheter insertion<br>o Opioid requirements post-discharge as assessed by prescriptions provided at time of discharge and during post-discharge follow-up visit using both patient health record and direct patient recounting<br>o Chronic post sternotomy pain/disability and patient reported outcomes assessed at 3 months ± 4 weeks from surgery with McGill Pain Questionnaire as well as at 6 weeks ± 2 weeks and 3 months ± 4 weeks with the change in PROMIS 29 score
Statistical Analysis	Categorical data will be summarized by frequencies and percentages, and continuous variables by means and standard deviations (or medians and interquartile ranges for non-linear data). For the primary analysis, we will perform linear regression, adjusted for sex and intraoperative opioid MME to assess the comparison between trial groups. A modified intention-to-treat analysis will be employed, including only patients who received the block, and excluding patients with prolonged intubation. Sensitivity analyses using complete intention-to-treat and per protocol populations will also be conducted.

2. Introduction and Background

Acute postoperative pain following cardiac surgery remains a prevalent and consequential problem, [1, 2] with the majority of patients experiencing moderate to severe pain during the perioperative period despite treatment with analgesic therapy [3, 4]. Beyond the short-term impact, significant pain in the days following surgery has been demonstrated to be an important predictor of chronic postoperative pain development [5-9], with Fletcher et al. demonstrating that a 30% increase in chronic pain incidence at 12 months occurs with a 10% increase in time spent in severe pain on postoperative day 1 [9]. Indeed, rates of chronic postsurgical pain have been reported as high as 35% at 3 months, 27% at 1 year, and 20% at 3 years postoperatively amongst cardiac surgical patients [10]. Furthermore, insufficiently managed pain has been linked to morbidity and mortality risk, with clinically important sequalae including cardiac, pulmonary, musculoskeletal, and metabolic complications, prolonged intubation and hospital stays, psychological consequences, as well as increased opioid consumption [11-14]. The consequent loss of productivity combined with the health-care costs leads to a significant toll on the healthcare system; in 2008 it was estimated that the annual cost of chronic pain conditions, including postoperative pain, was $560-635 billion in the U.S. alone [15, 16].

High-dose, short-acting opioid analgesics have been a cornerstone of both intraoperative anesthesia and early postoperative pain management amongst cardiac surgical patients [14, 17, 18]. However, the utilization of short-acting opioids comes at the risk of either inadequate pain control or serious adverse consequences due to the significant fluctuations in blood opioid levels [14, 19]. In particular, potential ramifications of high doses of opioids include respiratory depression, hyperalgesia, dependence/addiction, tolerance, ileus, constipation, and over-sedation [14, 18-20]. Furthermore, previous literature has shown that nearly 10% of post-cardiac surgery patients develop new persistent opioid use [21]. Numerous strategies have been investigated to simultaneously manage postoperative pain management and decrease opioid usage following cardiac surgical procedures; these goals remain important research priorities in this field.

To address the need for additional multimodal analgesic options for post-cardiac surgery patients, there has been growing interest in the role of nerve blocks, and particularly those intervening on the medial aspect of the thorax. Local anesthetic techniques have become a

mainstay of pain control in many surgical specialties [22], but there has been limited evidence definitively confirming their role in cardiac surgery. In one such technique, the superficial parasternal intercostal plane (PIP) block or the pectointercostal fascial plane block (PIFB), local anesthetic is deposited in a plane between the pectoralis major and intercostal muscles under ultrasound guidance to allow for visualization of the anesthetic spread [23, 24]. The anterior branches of the intercostal nerves are the major target for this block [25]. A recent review identified 10 randomized studies investigating the efficacy of parasternal intercostal nerve blocks in cardiac surgery patients, 8 of which specifically studied superficial PIP blocks, and none of which enrolled more than 100 patients [26]. While some centers have implemented these blocks and initial studies are promising for the ability of these interventions to reduce pain or opioid requirements [27-33], this reduction has not reached significance in every trial [29, 31], and there remains some conflict in the literature regarding the ability of these interventions to improve indices of respiratory performance [26, 28, 31, 33, 34].

One of the major limitations of nerve blocks administered via single doses of local anesthetic is their limited duration of action. For example, one prospective study of 113 participants undergoing cardiac surgery receiving ultrasound-guided deep PIP blocks with 40 mL 0.33% ropivacaine demonstrated a mean effective duration of a mere 17 hours [35]. One previous randomized study of 116 patients has investigated the role of continuous superficial PIP blocks in cardiac surgical patients, finding a significant reduction in time to extubation, time to mobilization, length of ICU stay, length of hospitalization, nausea/vomiting, pain scores at rest and with movement, and sufentanil/flurbiprofen consumption [36]. One other study investigated the potential for re-dosing of the superficial PIP block, randomizing 80 patients to ultrasound-guided superficial PIP block versus placebo on postoperative days 0 and 1. The authors demonstrated a reduction in pain scores, but were unable to show a significant reduction in opioid requirements over 48 hours nor incidence of delirium [29].

As such, there is still equipoise on the role of intermittent superficial PIP blocks in the multimodal approach to analgesia following cardiac surgery, and consequently guidelines are limited in the recommendations that can be provided on the use of such interventions [37]. The purpose of the present randomized trial is to definitively establish the clinical efficacy of

intermittent superficial PIP blocks with ropivacaine in optimizing postoperative pain control amongst a large population of patients undergoing cardiac surgery via median sternotomy.

3. Study Design

3.1 Study Overview

EPOCH CardioLink-10 is a randomized, double-blinded, parallel-group, multicentre study of superficial parasternal intercostal plane blocks with 0.2% ropivacaine versus sham block with 0.9% normal saline administered via catheters placed under ultrasound guidance in patients undergoing cardiac surgery through a median sternotomy. Patients meeting inclusion criteria who provide informed consent will be randomized in a 1:1 fashion into the intervention or control group. Based on the below sample size calculation, approximately 340 patients will need to be randomized to detect important differences. This will be a multicentre trial (up to 4 sites in Canada), with usual institutional anesthetic and postoperative analgesia management being provided to all enrolled patients. Patients will be followed prospectively during the hospitalization with regular assessments of postoperative pain and recovery, as well as during follow-up visits at 6 weeks and 3-6 months following their operation. To reduce lost-to-follow-up, patients will be asked to provide up to two telephone contact numbers.

The four Canadian trial sites are:

1) St. Michael's Hospital, Toronto, ON (Recruiting)
   PI: Dr. Ahmad Alli
2) QEII Health Sciences Centre, Halifax, NS (Recruiting)
   PIs: Dr. James Dougherty, Dr. Pieter de Jaeger
3) Foothills Medical Centre, Calgary, AB (Recruiting)
   PI: Dr. Christopher Noss; Co-I: Dr. Alexander Gregory
4) Royal Columbian Hospital, New Westminster, BC (Recruiting)
   PI: Dr. Pawel Martinka; Co-I: Korey Sutherland, MN

3.2 Primary Endpoint

- Cumulative postoperative opioid use measured as Milligram Morphine Equivalent (MME) up to 72 hours following catheter insertion, calculated based on the following conversion table [38]

Opioids	Conversion Factor
**Oral Opioids**	
Codeine (mg)	0.15
Tramadol (mg)	0.1
Hydrocodone (mg)	1
Oxycodone (mg)	1.5
Methadone (mg/day)	
1-20	4
21-40	8
41-60	10
≥61-80	12
Morphine (mg)	1
Hydromorphone (mg)	4
**Transdermal Opioids**	
Fentanyl (**μ**g/hour)	2.4
**Intravenous Opioids**	
Morphine (mg)	3
Hydromorphone (mg)	15
Fentanyl (**μ**g)	0.2

3.3 Secondary Endpoints

- Median pain score as measured by a standardized numeric rating scale over 72 hours following catheter insertion (measured at rest and with coughing q12hours ± 2 hours post-catheter insertion)
- Cumulative postoperative opioid use measured as Milligram Morphine Equivalent (MME) from catheter insertion until discharge from hospital

- Assessment for delirium BID for 72 hours following catheter insertion, as defined by the presence of one of the following criteria: Confusion Assessment Method (CAM) or CAM-ICU positive, or Intensive Care Delirium Screening Checklist (ICDSC) >3, or 3D-CAM, or 4AT ≥4, or more than one dose of haloperidol or similar antipsychotic drug, or documented delirium by neurologist or psychiatrist consultation
- Quality of Recovery-15 Scale administered 24-96 hours post-surgery

3.4 Tertiary (exploratory) Endpoints

- Time from catheter insertion to extubation
- Time from catheter insertion to first opioid analgesic provision following extubation
- Time from catheter insertion to mobilization
- ICU and hospital LOS
- Postoperative nausea and vomiting, measured via a 10-point visual analogue scale BID and antiemetic medication requirements up to 72 hours following catheter insertion
- Opioid requirements post-discharge as assessed by prescriptions provided at time of discharge and during post-discharge follow-up visit using both patient health record and direct patient recounting
- Chronic post sternotomy pain/disability and patient reported outcomes assessed at 3 months ± 4 weeks from surgery with McGill Pain Questionnaire as well as at 6 weeks ± 2 weeks and 3 months ± 4 weeks with the change in PROMIS 29 score (Attempts to obtain the 3-month data can continue for up to 6 months post-surgery before it will be considered a protocol deviation or the participant deemed lost-to-follow-up)

3.5 Randomization Procedure

Randomization will be performed via a centralized, web-based platform that uses permuted blocks of varying sizes. Randomization will be stratified by site and patient sex. Dedicated unblinded individuals at each site who are responsible for preparing the assigned blinded bags of ropivacaine and saline will be provided with a randomization code list. Details on participant allocation and the randomization code lists will not be shared with the blinded study team members.

3.6 Study Interventions

All patients will have their cardiac surgery and anesthesia as per standard of care. Patients assigned to either group will undergo administration of general anesthesia, endotracheal intubation, vascular access procedures, and transesophageal echocardiography probe insertion as per usual cardiac anesthesia practice. Usual anesthetic monitoring practice will continue before, during, and after the block is performed.

Catheter Insertion

Catheter insertion will be performed by an anesthesiologist with specialized regional block training. Catheters (Arrow® or PAJUNK®) will be inserted under ultrasound guidance, in a sterile fashion, with a high-frequency linear transducer which will be placed 1 cm lateral to the sternal border in the longitudinal plane in order to view the T4-T5 intercostal space. A 16 or 17-gauge Tuohy needle (or alternate catheter suitable nerve block needle) will be used to enter the superficial PIP, with saline being injected to help visualize the plane between the pectoralis major and intercostal muscles. Subsequently, the catheters will be advanced into the plane and secured on the skin.

Study Intervention Administration:

Bags of 0.2% ropivacine and 0.9% saline will only be identifiable by the participants' unique study numbers on the labels and transported to the OR or ICU in UV light-resistant bags. The bags of ropivacine and saline, still in their UV light-resistant bags will be set up in nerve block infusion pumps (e.g. CADD Solis [ICUMedical, San Clemente, California]) for administration. Following catheter insertion, the bags of ropivacaine and saline will be attached via dedicated tubing to each study drug administration catheter (one pump per catheter). An initial bolus of 20 mL per side of study intervention will be administered through the pumps. Subsequently, a basal infusion rate (0.1-1 mL/hr per side) will be initiated and will continue for 48 hours, with an additional 5 mL bolus every 3-4 hours for 48 hours. This will result in a total of 85-148 mL of study intervention per side (170-296 mL total) being administered to each patient during the study. This equates to a maximum of 592 mg of ropivacaine administered during the 48-hour drug administration period. This is well within accepted safe limits as described in the product monograph [39].

3.7 Blinding

The participants, clinical team, intervention appliers (i.e. the operating room staff), outcome assessors, and data entry support team will be blinded to the randomized treatment arm until the database is locked. Blinded study bags will be inserted into the infusion pumps. Unblinding procedures are available if necessary for clinical treatment e.g. cases of suspected local anesthetic systemic toxicity (LAST) or allergic reactions. Site investigators should contact the PI of the lead site (Dr. Ahmad Alli) to request the code break. The date and reason(s) for breaking the blind must be recorded in the source documentation and electronic case report forms (eCRFs), as applicable.

4. Eligibility Criteria

4.1 Inclusion Criteria

1. Adult patients undergoing cardiac surgery via median sternotomy

4.2 Exclusion Criteria

1. Redo sternotomy, or cardiac surgery performed through non-sternotomy approaches (minimally invasive procedures, thoracotomies, mini-sternotomy, hemi-sternotomy, etc.)
2. Emergency procedures (surgery within 2 hours)
3. Clinical instability which in the judgement of the investigator precludes enrollment or participation in the study.
4. Weight < 50 kg
5. Active systemic bacterial infection including infective endocarditis or pre-existing sternal infections
6. Surgery for infective endocarditis
7. Pregnancy or nursing
8. Chronic opioid/narcotic use > 6 weeks, active use of illicit drugs, long-term opioid exposure or opioid use and/or chronic pain disorder/syndromes
9. Allergies to amide anesthetic agents or any components of study interventions
10. Inability to comply with, or participate in, protocol (i.e. cognitive impairment/altered mental status/neurological deficit or disorder, inability to provide informed consent, inability to complete pain rating scales, etc.)
11. Receipt of an investigational drug or device within past 7 days

5. Study Procedure and Visits

5.1 Summary of Visits

Patients will be screened at an initial preoperative visit at which point eligibility will be determined. Patient outcomes will be assessed throughout their postoperative hospital stay as detailed above. Two follow-up telephone visits after the operation will be scheduled. The first will be at 6 weeks ± 2 weeks post surgery and the second will be at 3 months ± 4 weeks.

5.2 Study Visits

Visit S: Screening

- Written informed consent
- Determination of eligibility
- Demographics, medical history, baseline medications including doses
- Physical examination, height and weight assessment, BMI
- Vital signs
- Laboratory assessments: CBC, creatinine/eGFR, electrolytes, coagulation testing
- Preoperative PROMIS 29 Score

Visit R: Preoperative Randomization

- Confirmation of eligibility
- Randomization

Hospitalization

- Cumulative postoperative opioid use measured as Milligram Morphine Equivalent (MME) from catheter insertion until discharge from hospital, obtained via medication administration record (MAR) in patients' health record.
- Median pain score as measured by a standardized numeric rating scale over 72 hours following catheter insertion (measured at rest and with coughing q12hours ± 2 hours post-catheter insertion). First assessment will be conducted when patient is awake and able to follow commands following extubation, second assessment will be conducted at the first intervention dose following first assessment, and ongoing assessments will be conducted at approximately every 12 hours thereafter.

- Delirium assessed BID for 72 hours following catheter insertion (see above criteria). First assessment will be conducted when patient is awake and able to follow commands following extubation, second assessment will be conducted at the first intervention dose following first assessment, and ongoing assessments will be conducted at approximately every 12 hours thereafter.
- Time from catheter insertion to extubation
- Time from catheter insertion to first opioid analgesic provision following extubation
- Time from catheter insertion to mobilization
- ICU and hospital LOS
- Postoperative nausea and vomiting, measured via a 10-point visual analogue scale BID and antiemetic medication requirements up to 72 hours following catheter insertion. First assessment will be conducted when patient is awake and able to follow commands following extubation, second assessment will be conducted at the first intervention dose following first assessment, and ongoing assessments will be conducted at approximately every 12 hours thereafter.
- Quality of Recovery-15 Scale administered 24-96 hours post-surgery

Visit P1: Postoperative Follow-Up: 6 weeks ± 2 weeks post surgery (Phone-Call Visits)

- PROMIS 29 score
- Opioid requirements post-discharge as assessed by prescriptions provided at time of discharge and during post-discharge follow-up visit using both patient health record and direct patient recounting

Visit P2: Postoperative Follow-Up: 3 months ± 4 weeks post surgery (Phone-Call Visit)

- PROMIS 29 score
- McGill Pain Questionnaire
- Opioid requirements post-discharge as assessed by prescriptions provided at time of discharge and during post-discharge follow-up visit using both patient health record and direct patient recounting
- Attempts to obtain the 3-month data can continue for up to 6 months post-surgery before it will be considered a protocol deviation or the participant deemed lost-to-follow-up

6. Subject Safety Monitoring and Adverse Events

Risks associated with the proposed intervention include: infection (both soft tissue and intravascular), hematoma/bleeding, vascular injury, pneumothorax/hemothorax and allergic reaction/anaphylaxis [24]. In an investigation of intermittent bolus erector spinae plane blocks in cardiac surgical patients receiving lateral mini-thoracotomy, adverse events included: pneumothorax (~1%), staxis around stoma (~5%, generally less than 10 mL), hypotension (~1%), catheter displacement (~3%), and catheter obstruction secondary to clotting (~2%) [40]. The most common adverse effects to ropivacaine specifically are: hypotension (32%), nausea (17%), vomiting (7%), bradycardia (6%), and headache (7%) [41]. Furthermore, local anesthetic systemic toxicity (LAST) occurs at a rate of approximately 1.8 in 1000 local anesthetic administrations [42]. Signs and symptoms generally manifest within 1-5 minutes of administration and include oral numbness, metallic taste, dizziness, drowsiness and disorientation; more severe manifestations can appear up to 6 hours after initial symptoms and may include seizures, arrythmias, hypotension, cardiac arrest and death. Continuous blockade via catheter delivery of local anesthetic may increase risk of LAST compared with single-shot techniques, although ropivacaine is associated with less cardiotoxicity than other local anesthetics [43, 44]. In addition, patients with cardiac disease are at an increased risk of LAST. In particular, those with conduction disorders may have a higher risk of cardiotoxicity. As such, the use of less cardiotoxic drugs like ropivacaine is recommended for these patients [44]. Ropivacaine also has a higher cardiovascular collapse/CNS ratio compared with racemic bupivacaine, and therefore has a theoretical safety advantage for long-acting local anesthetic purposes [44]. Nevertheless, current evidence suggests PIF blocks are safe and easy to perform, especially considering the more superficial location of the plane (and therefore the increased distance from vital structures) compared with a deep PIP or transversus thoracis muscle plane block [24].

In the present investigation, each study visit will include safety monitoring. Adverse effects that will be specifically collected include infection, hematoma/hemorrhage, vascular injury, pneumothorax/hemothorax, allergic reaction/anaphylaxis, postoperative nausea/vomiting, pruritus and LAST. Adverse event data will be reported in the study publication.

6.1 Data and Safety Monitoring Board (DSMB)

The Data and Safety Monitoring Board (DSMB) will review safety reports after 50% of the trial population has completed follow-up and can, at their discretion, request additional safety analyses. The DSMB will make recommendations as it sees fit regarding the conduct and continuation/stopping of the trial to ensure the safety of the participants and the integrity of the trial.

7. Statistical Analysis and Sample Size Calculation

For the primary analysis, we will perform linear regression, adjusted for sex (clinical stratification factor) and intraoperative opioid MME to assess the comparison between trial groups, with a P-value threshold of 0.05 being considered significant. If residuals indicate substantial violations of the linear regression assumptions, alternate methods will be attempted, such as analyzing log-transformed data, using bootstrap hypothesis testing or two-sample t-tests without equal variance. A modified intention-to-treat analysis will be employed, including only patients in whom a block catheter was placed, and a dose of study drug was administered. The time-to-event outcomes will be modelled simultaneously using a multistate framework. This approach will handle competing risks, such as death, and treat other non-fatal events, that could be related to each other, as intercurrent events. Sensitivity analyses using complete intention-to-treat and per protocol populations will also be conducted. Categorical data will be summarized by frequencies and percentages, and continuous variables by means and standard deviations (plus medians and interquartile ranges for non-symmetric data).

In order to detect a mean difference of 10 MMEs, assuming a standard deviation of 25 and an alpha value of 0.05, a sample size of 133 participants per group would be required to provide a power of 90%. Assuming an attrition rate of 25%, approximately 340 patients will need to be randomized for the primary modified intention-to-treat analysis.

Pre-specified subgroup analyses of the primary outcome include:

- Age
- Race/Ethnicity
- Sex/Gender
- Diabetes status
- BMI
- Type of operation
- Site

8. Data Collection and Storage

Trial Management

The Applied Health Research Centre (AHRC) of St. Michael's Hospital will be coordinating centre for the study. The AHRC will provide project and data management, as well as trial monitoring. The AHRC is a comprehensive clinical trials unit and is the official clinical trials unit for the University of Toronto. REDCap will be utilized for randomization and eCRF data entry. REDCap is a web-based electronic data capture system with built-in quality checks to ensure data accuracy and integrity.

Investigator Responsibilities and Obligations

The protocol will be adhered to in conducting this trial, as will the GCP and applicable regulatory requirement(s). The investigator may decide to implement protocol deviations where it protects safety, wellbeing and/or is in the best interest of the participant (e.g. if the investigator determines in-person visits increase risk of participants contracting COVID-19).

Data Management Responsibilities

As aforementioned, REDCap will be used for this trial; all CRF data will be entered in electronic forms at the investigator site. Authorized study site personnel, designated by the study investigator, will complete the data collection process. Appropriate training and security measures will be completed with the site investigator and all authorized study site personnel. Data will be housed at the AHRC at St. Michael's Hospital in Toronto, ON, Canada throughout the duration of the study.

Source Documents and Access

Appropriate medical and research records for this trial will be maintained by the study team. The study team will comply with requirements laid out by the relevant regulatory/institutional bodies for the protection of patients' confidentiality. The data collected on the eCRFs will be supported by source documentation. The site principal investigator will be responsible for making sure that the data collected are complete, accurate, and recorded in a timely manner. Access to source documentations will be determined based on regulatory and institutional guidelines. All study records will be kept for 7 years.

References

1. Mueller XM, Tinguely F, Tevaearai HT, et al. Pain location, distribution, and intensity after cardiac surgery. Chest. 2000;118(2):391-6.
2. Milgrom LB, Brooks JA, Qi R, et al. Pain levels experienced with activities after cardiac surgery. Am J Crit Care. 2004;13(2):116-25.
3. Gelinas C. Management of pain in cardiac surgery ICU patients: have we improved over time? Intensive Crit Care Nurs. 2007;23(5):298-303.
4. Lahtinen P, Kokki H, Hynynen M. Pain after cardiac surgery: a prospective cohort study of 1-year incidence and intensity. Anesthesiology. 2006;105(4):794-800.
5. Chidambaran V, Ding L, Moore DL, et al. Predicting the pain continuum after adolescent idiopathic scoliosis surgery: A prospective cohort study. Eur J Pain. 2017;21(7):1252-65.
6. Althaus A, Arranz Becker O, Moser KH, et al. Postoperative Pain Trajectories and Pain Chronification-an Empirical Typology of Pain Patients. Pain Med. 2018;19(12):2536-45.
7. Bayman EO, Parekh KR, Keech J, et al. A Prospective Study of Chronic Pain after Thoracic Surgery. Anesthesiology. 2017;126(5):938-51.
8. Gilron I, Vandenkerkhof E, Katz J, et al. Evaluating the Association Between Acute and Chronic Pain After Surgery: Impact of Pain Measurement Methods. Clin J Pain. 2017;33(7):588-94.
9. Fletcher D, Stamer UM, Pogatzki-Zahn E, et al. Chronic postsurgical pain in Europe: An observational study. Eur J Anaesthesiol. 2015;32(10):725-34.
10. Marcassa C, Faggiano P, Greco C, et al. A retrospective multicenter study on long-term prevalence of chronic pain after cardiac surgery. J Cardiovasc Med (Hagerstown). 2015;16(11):768-74.
11. Cogan J. Pain management after cardiac surgery. Semin Cardiothorac Vasc Anesth. 2010;14(3):201-4.
12. Puntillo K, Weiss SJ. Pain: its mediators and associated morbidity in critically ill cardiovascular surgical patients. Nurs Res. 1994;43(1):31-6.
13. Roediger L, Larbuisson R, Lamy M. New approaches and old controversies to postoperative pain control following cardiac surgery. Eur J Anaesthesiol. 2006;23(7):539-50.
14. Iguidbashian JP, Chang PH, Iguidbashian J, et al. Enhanced recovery and early extubation after pediatric cardiac surgery using single-dose intravenous methadone. Ann Card Anaesth. 2020;23(1):70-4.
15. Relieving Pain in America: A Blueprint for Transforming Prevention, Care, Education, and Research. The National Academies Collection: Reports funded by National Institutes of Health. Washington (DC)2011.
16. Gan TJ. Poorly controlled postoperative pain: prevalence, consequences, and prevention. J Pain Res. 2017;10:2287-98.
17. Bovill JG, Sebel PS, Stanley TH. Opioid analgesics in anesthesia: with special reference to their use in cardiovascular anesthesia. Anesthesiology. 1984;61(6):731-55.
18. Robinson JD, Caruso TJ, Wu M, et al. Intraoperative Methadone Is Associated with Decreased Perioperative Opioid Use Without Adverse Events: A Case-Matched Cohort Study. J Cardiothorac Vasc Anesth. 2020;34(2):335-41.
19. Murphy GS, Szokol JW, Avram MJ, et al. Intraoperative Methadone for the Prevention of Postoperative Pain: A Randomized, Double-blinded Clinical Trial in Cardiac Surgical Patients. Anesthesiology. 2015;122(5):1112-22.

20. Barletta JF. Clinical and economic burden of opioid use for postsurgical pain: focus on ventilatory impairment and ileus. Pharmacotherapy. 2012;32(9 Suppl):12S-8S.
21. Brown CR, Chen Z, Khurshan F, et al. Development of Persistent Opioid Use After Cardiac Surgery. JAMA Cardiol. 2020;5(8):889-96.
22. Rawal N. Current issues in postoperative pain management. Eur J Anaesthesiol. 2016;33(3):160-71.
23. Hong B, Oh C, Jo Y, et al. Current evidence of ultrasound-guided fascial plane blocks for cardiac surgery: a narrative literature review. Korean J Anesthesiol. 2022;75(6):460-72.
24. Kelava M, Alfirevic A, Bustamante S, et al. Regional Anesthesia in Cardiac Surgery: An Overview of Fascial Plane Chest Wall Blocks. Anesth Analg. 2020;131(1):127-35.
25. Sepolvere G, Coppolino F, Tedesco M, Cristiano L. Ultrasound-guided parasternal blocks: techniques, clinical indications and future prospects. Minerva Anestesiol. 2021;87(12):1338-46.
26. Schiavoni L, Nenna A, Cardetta F, et al. Parasternal Intercostal Nerve Blocks in Patients Undergoing Cardiac Surgery: Evidence Update and Technical Considerations. J Cardiothorac Vasc Anesth. 2022;36(11):4173-82.
27. Bloc S, Perot BP, Gibert H, et al. Efficacy of parasternal block to decrease intraoperative opioid use in coronary artery bypass surgery via sternotomy: a randomized controlled trial. Reg Anesth Pain Med. 2021;46(8):671-8.
28. Hamed MA, Abdelhady MA, Hassan A, Boules ML. The Analgesic Effect of Ultrasound-guided Bilateral Pectointercostal Fascial Plane Block on Sternal Wound Pain After Open Heart Surgeries: A Randomized Controlled Study. Clin J Pain. 2022;38(4):279-84.
29. Khera T, Murugappan KR, Leibowitz A, et al. Ultrasound-Guided Pecto-Intercostal Fascial Block for Postoperative Pain Management in Cardiac Surgery: A Prospective, Randomized, Placebo-Controlled Trial. J Cardiothorac Vasc Anesth. 2021;35(3):896-903.
30. Kumar AK, Chauhan S, Bhoi D, Kaushal B. Pectointercostal Fascial Block (PIFB) as a Novel Technique for Postoperative Pain Management in Patients Undergoing Cardiac Surgery. J Cardiothorac Vasc Anesth. 2021;35(1):116-22.
31. Krishnan S, Desai R, Paik P, et al. Superficial Parasternal Intercostal Plane Blocks (SPIB) With Buprenorphine, Magnesium, and Bupivacaine for Management of Pain in Coronary Artery Bypass Grafting. Cureus. 2022;14(11):e30964.
32. Dost B, De Cassai A, Balzani E, et al. Effects of ultrasound-guided regional anesthesia in cardiac surgery: a systematic review and network meta-analysis. BMC Anesthesiol. 2022;22(1):409.
33. Zhang Y, Gong H, Zhan B, Chen S. Effects of bilateral Pecto-intercostal Fascial Block for perioperative pain management in patients undergoing open cardiac surgery: a prospective randomized study. BMC Anesthesiol. 2021;21(1):175.
34. Li J, Lin L, Peng J, et al. Efficacy of ultrasound-guided parasternal block in adult cardiac surgery: a meta-analysis of randomized controlled trials. Minerva Anestesiol. 2022;88(9):719-28.
35. Zhang Y, Min J, Chen S. Sensory Assessment and Block Duration of Deep Parasternal Intercostal Plane Block in Patients Undergoing Cardiac Surgery: A Prospective Observational Study. Pain Ther. 2022;11(3):951-8.
36. Zhang Y, Min J, Chen S. Continuous Pecto-Intercostal Fascial Block Provides Effective Analgesia in Patients Undergoing Open Cardiac Surgery: A Randomized Controlled Trial. Pain Med. 2022;23(3):440-7.

37. Engelman DT, Ben Ali W, Williams JB, et al. Guidelines for Perioperative Care in Cardiac Surgery: Enhanced Recovery After Surgery Society Recommendations. JAMA Surg. 2019;154(8):755-66.
38. Harvin JA, Green CE, Vincent LE, et al. Multi-modal Analgesic Strategies for Trauma (MAST): protocol for a pragmatic randomized trial. Trauma Surg Acute Care Open. 2018;3(1):e000192.
39. Ltd FKC. PRODUCT MONOGRAPH Ropivacaine Hydrochloride Injection, USP. 2018.
40. Sun Y, Luo X, Yang X, et al. Benefits and risks of intermittent bolus erector spinae plane block through a catheter for patients after cardiac surgery through a lateral mini-thoracotomy: A propensity score matched retrospective cohort study. J Clin Anesth. 2021;75:110489.
41. George AM, Liu M. Ropivacaine. StatPearls. Treasure Island (FL)2022.
42. Macfarlane AJR, Gitman M, Bornstein KJ, et al. Updates in our understanding of local anaesthetic systemic toxicity: a narrative review. Anaesthesia. 2021;76 Suppl 1:27-39.
43. Antel R, Ingelmo P. Local anesthetic systemic toxicity. CMAJ. 2022;194(37):E1288.
44. El-Boghdadly K, Pawa A, Chin KJ. Local anesthetic systemic toxicity: current perspectives. Local Reg Anesth. 2018;11:35-44.

EPOCH CardioLink-10 Randomized Clinical Trial
REB Amendment Summary of Changes

Summary of Changes Made	Explanation for Change
Changes Made to Informed Consent Form	
Study Coordinator changed from Nilou Siadati to Kyle Chin (contact information changed accordingly)	Staffing changes made to project
Assessment of nausea/vomiting and delirium have been indicated in the study procedure table as being performed for research purposes only. In addition, the table has been updated to reflect that pain assessments, delirium assessments, and nausea/vomiting assessments will be conducted approximately twice per day.	This change has been made as objective mechanisms for measuring nausea/vomiting and delirium have been added to the protocol, which are outside the scope of regular clinical care/practice. The addition of the frequency of assessments have been included for patients' information in accordance with the protocol.
Post-discharge phone visit expected time changed from 10 minutes to 30 minutes and assessment of pain medication requirements since surgery has been added to the study procedure table under the follow-up phone call visit column.	Following further evaluation of the questionnaires being used during the post-discharge visit, in addition to the inclusion of post-discharge opioid requirements as an exploratory outcome for this study that will require assessment during this phone visit, the length of time that will be required for this visit has been extended.
A page for consent to be documented with a declaration of assistance from a language interpreter has been added	In order to ensure our study is accessible as possible and that our study population reflects the population we serve, we have included a mechanism for patients with limited English proficiency to still participate. In particular, we plan on using the virtual translation service that is already in place at SMH to allow for a comprehensive consent conversation to take place prior to a patient deciding if they would like to participate. The same service would be used throughout the study for the necessary evaluations/study procedures.
Patient questionnaires in the study procedure table and corresponding text sections of the ICF have been updated to reflect the below changes to the protocol including:<br>• Administration of the Recovery Questionnaire 24-96h post-surgery	These changes have been made in accordance with recommendations from experts in the field.

• Administration of the PROMIS 29 questionnaire for patient-reported outcome measures at baseline and at 3 months postoperatively<br>• Removal of the WHODAS assessment	
Changes Made to Clinical Trial Protocol	
Change to secondary endpoints:<br><br>1. The endpoint of median pain score over 72 hours following extubation has been changed to over 72 hours following catheter insertion<br>2. The secondary endpoint of median pain score "during movement" has been changed to "with coughing"<br>3. Assessment of delirium using "institutional score" has been further explained with specific criteria including: "Confusion Assessment Method (CAM) or CAM-ICU positive, or Intensive Care Delirium Screening Checklist (ICDSC) >3, or 3D-CAM, or 4AT ≥4, or more than one dose of haloperidol or similar antipsychotic drug, or documented delirium by neurologist or neurosurgeon or psychiatrist consultation"<br>4. Quality of Recovery-15 scale has been changed from an exploratory endpoint to a secondary endpoint, and will be administered 24-96 hours post-surgery	1. This change has been made to ensure a standardized duration of assessment for all outcomes (72 hours post-catheter insertion)<br>2. In order to standardize the method by which we collect pain scores with movement, the protocol has been updated such that pain scores will be collected at rest and with coughing. This has been chosen as a result of methodology used in previous pain-related studies in cardiac surgery, as well as the wide generalizability of this methodology with post-cardiac surgery patients (i.e. some patients may be unable to mobilize or perform more complex movements).<br>3. The criteria used for determining the presence of delirium has been clarified to allow for an objective evaluation by outcome assessors. The criteria has been based off of previous studies evaluating delirium.<br>4. While this does not make any changes to the statistical analysis plan, this change has been made as a result of the increasingly significant role patient-reported quality of life and recovery is playing in evidence-based medical practices. The change to administration within 24-96 hours post-surgery is based off input from experts in the field.
Changes made to tertiary (exploratory) endpoints<br><br>1. "Time to first analgesic request" has been changed to "time from catheter insertion to first opioid analgesia provision following extubation"<br>2. Assessment of postoperative nausea and vomiting now also includes antiemetic	1. Given that analgesia provisions are documented more reliably than requests for pain medication, we have altered this outcome to ensure accurate reporting of this outcome.<br>2. We have added this element to the postoperative nausea and vomiting outcome given that scores may be artificially low if a patient is receiving antiemetic medications

medication requirements up to 72 hours following catheter insertion<br>3. An additional outcome assessing post-discharge opioid requirements has been added<br>4. A time window has been added for the post-discharge assessment<br>5. Patient questionnaires administered at the 3 month visit will now include the Montreal Pain Questionnaire and the PROMIS 29 score (the latter of which will also be administered at baseline). The WHODAS will no longer be administered.	3. An important exploratory outcome for this study is whether modulating patients' perioperative pain experience impacts the development of chronic post-sternotomy pain syndromes; this may also manifest as changes in post-discharge opioid requirements, which will now be assessed in this investigation.<br>4. Given that patient and outcome assessor schedules may make it difficult for the post-discharge phone call to be exactly 3 months following surgery, we have added a time window around this period during which time chronic post sternotomy pain/disability can still be assessed.<br>5. Changes the patient questionnaires have been made based off input from experts in the field.
A 17-gauage needle will be used in lieu of a 22-gauage needle for the purposes of entering the superficial PIP during catheter insertion	This modification has been made based on further clarification of the equipment required for the proposed blocks.
The dosing regimen for both placebo and intervention groups has been changed to q6-8 hours for 48 hours total	This change has been made to allow for standardization across all patients. In the previous iteration of the protocol, patients would have the dosing of study medications terminated once they are discharged from ICU – for some uncomplicated patients, this may be a relatively short period and therefore they may receive a very small number of doses, therefore confounding the final results. As such, we have now developed a mechanism by which study drug can be provided on the ward, and therefore we have standardized the regimen such that all patients will receive doses for a total of 48 hours.
Changes made to Exclusion criteria<br><br>1. Exclusion criteria related to substance abuse and chronic opioid use have been combined into the current exclusion criteria #8, which is focused on chronic opioid/narcotic use<br>2. Requirement of preoperative IABP support has been removed as an exclusion criteria for this study.<br>3. Surgery for infective endocarditis has been added as an exclusion criteria	 <br>1. This exclusion criteria has been updated to focus specifically on the patients for whom their history may preclude adequate assessment of the intervention being proposed in this study, and for whom the addition of these medications may impact their safety.<br>2. It is not uncommon for patients to require IABP support prior to cardiac surgery, and thus to exclude all such patients could limit the generalizability. In addition, a "clinical instability" exclusion criteria is included, and

	thus if it is unsafe or infeasible to proceed with the study protocol, these patients would be excluded through this criteria anyway.<br>3. Previously thought to be included under the exclusion criteria related to active systemic bacterial infections, this exclusion criteria has been added to provide absolute clarity that patients undergoing surgery for infective endocarditis should be excluded, as those patients have a different perioperative course that may preclude from safe participation in this study as well as adequate assessment of the efficacy of the proposed intervention.
Preoperative WHODAS score assessment added to screening visit	This amendment has been made so patients' preoperative disability status is documented for the purposes of better understanding relative benefit of the intervention.
Changes made to time point assessments for pain, delirium, and postoperative nausea and vomiting.	These changes have been made to provide more clarity to outcome assessors as to when to conduct assessments, and also to improve study protocol adherence by reducing the interruption and impact to both patients and providers through combining multiple study procedures including medication provision, pain assessment, delirium assessment, and nausea/vomiting assessment.

EPOCH CardioLink-10 Randomized Clinical Trial
REB Amendment Summary of Changes

Summary of Changes Made	Explanation for Change
1) Study Intervention Delivery<br><br>Study intervention (either placebo or ropivacaine) will now be delivered by Continuous Ambulatory Delivery Device (CADD) pumps which have recently been acquired at SMH. There will still be an initial bolus of 20mL/side at the time of catheter insertion; this will be followed by a basal continuous infusion of (0.1-1 mL/hr/side and 5mL/side boluses q3 to q4hrs for 48 hours following catheter insertion(total volume of 200-240 mL over the study period of either placebo or intervention).	SMH has recently acquired CADD pumps for use with all types of regional anesthesia blocks as standard of care. This change has also been made to reduce study personnel burden by having the study intervention given by a standard clinical delivery system. Furthermore, this change should improve standardization and consistency of study intervention delivery time and volume, thus improving the internal validity and reproducibility of our results.
2) PROMIS-29 Assessment will be performed at baseline, 6 weeks and 3 months	The addition of the PROMIS-29 assessment at 6 weeks has been done for two central reasons. First, it will provide more informative data on short-term effects of the study intervention on patient reported outcome measures, and thus any differences at this time may be more directly attributable to the study interventions. Second, it will also allow a time trend of patient reported outcome measures to be determined (from baseline to 6 weeks to 3 months), which may provide important mechanistic insights into how the study interventions may result in differing outcomes.

Summary of Changes

Study Title: Efficacy of Superficial Parasternal Intercostal Plane Block on Postoperative Pain Control in Patients Undergoing Cardiac Surgery with Median Sternotomy [EPOCH CardioLink-10]

PI: Ahmad Alli

REB Number: 23-038

Replicate the table below as many times as needed to describe each change.

**Change #1**	Clarified that this is a double-blinded, multicentre trial
**Rationale:**	Reduce bias and improve generalizability of the trial results
**Risk:**	No Risk
**Impact:** (e.g. Participant safety, study outcomes, study budget)	No impact on participant safety, study outcomes or study budget
**Impacted Documents:**	Protocol, ICF

**Change #2**	Increase in total sample size from 310 to 314
**Rationale:**	Increase statistical power to detect a meaningful difference between the intervention and the control
**Risk:**	No Risk
**Impact:**	No impact on participant safety, study outcomes or study budget<br>Enrollment target at SMH will remain capped at 270
**Impacted Documents:**	Protocol, ICF

**Change #3**	Request for a second telephone contact number
**Rationale:**	To reduce lost-to-follow-up due to inability to reach participants at a single contact number
**Risk:**	No Risk
**Impact:**	No impact on participant safety, study outcomes or study budget
**Impacted Documents:**	Protocol, ICF

**Change #4**	Allowance for the second telephone visit to be conducted up to 6 months post-surgery after which a protocol deviation needs to be reported
**Rationale:**	To increase flexibility and reduce lost-to-follow-up (e.g. due to work commitments and travel for work or during school, winter and summer holidays)
**Risk:**	No Risk - the available literature indicates that participant reported outcomes collected up to 6 months after intervention remain valid and can provide meaningful insights
**Impact:**	No impact on participant safety; Limited impact on study budget (longer study duration requires more resources); Potential impact on study outcomes and data analyses/interpretation given the wider follow-up window
**Impacted Documents:**	Protocol, ICF

**Change #5**	Stratification of randomization by site and patient sex
**Rationale:**	Ensure better balance between groups
**Risk:**	No Risk
**Impact:**	No impact on participant safety, study outcomes or study budget
**Impacted Documents:**	Protocol

**Change #6**	Included details of other participating sites in Canada and their lead investigators
**Rationale:**	Enhance generalizability of study findings (4 sites with different healthcare settings and participant pools in 4 provinces – NS, ON, AB and BC)
**Risk:**	No Risk
**Impact:**	No impact on participant safety, study outcomes or study budget
**Impacted Documents:**	Protocol

**Change #7**	Removed neurosurgeons as possible assessors of delirium
**Rationale:**	Neurosurgeons are never involved in assessment of delirium in CVICU patients. This is done by neurologists or psychiatrists (who are still included in protocol).
**Risk:**	No Risk
**Impact:**	No impact on participant safety, study outcomes or study budget
**Impacted Documents:**	Protocol

**Change #8**	Expanded description of the randomization, IP preparation and IP administration procedures
**Rationale:**	The sites have slightly different procedures due to different local SOPs. All sites have a limited number of individuals who are unblinded to study group assignment to allow for IP preparation and dispensing to blinded colleagues. Team members who administer the allocated IP will remain blinded for the course of the trial to avoid bias and increase validation of the study design and data.
**Risk:**	No Risk
**Impact:**	No impact on participant safety, study outcomes or study budget with improved generalizability
**Impacted Documents:**	Protocol

**Change #9**	Expanded details on types of catheters and needles use for IP delivery
**Rationale:**	Sites have different budgets, vendors and vendor contracts
**Risk:**	No Risk
**Impact:**	No impact on participant safety, study outcomes, study budget, or IP delivery
**Impacted Documents:**	Protocol

**Change #10**	Expanded on circumstances and procedures for breaking the blind
**Rationale:**	Ensure participant safety
**Risk:**	Low Risk
**Impact:**	Ensure participant safety; study outcome reports may be affected
**Impacted Documents:**	Protocol

**Change #11**	Added description of DSMB
**Rationale:**	Clarifies safety monitoring process of the study
**Risk:**	No Risk
**Impact:**	Enhance patient safety
**Impacted Documents:**	Protocol, DSMB Charter

Change #12	Expanded description of planned statistical analyses
Rationale:	Clarifies planned analyses including addition of subgroup analyses
Risk:	None
Impact:	Improved description of data analysis and outcomes
Impacted Documents:	Protocol

Change #13	Staff Changes
Rationale:	Samson Moses is no longer at UHT; Adrian Quan is not participant facing; Kyle Chin will occasionally be participant facing
Risk:	No Risk
Impact:	No impact on participant safety, study outcomes or study budget
Impacted Documents:	ICF

Change #14	Inserted clinicaltrials.gov NCT reference number
Rationale:	Allows participants and likeminded investigators to become aware of the ongoing trial, its design, goals, and status
Risk:	No Risk
Impact:	No impact on participant safety, study outcomes or study budget
Impacted Documents:	ICF

Change #15	Changes to research team contact numbers
Rationale:	Streamline and improve the research participant-research team interaction
Risk:	No Risk
Impact:	No impact on participant safety, study outcomes or study budget
Impacted Documents:	ICF

Change #16	Corrected grammatical and typographical errors as well as formatting
Rationale:	To improve clarity and alignment between the protocol and ICF
Risk:	None
Impact:	None
Impacted Documents:	Protocol, ICF

Summary of Changes

Study Title: Efficacy of Superficial Parasternal Intercostal Plane Block on Postoperative Pain Control in Patients Undergoing Cardiac Surgery with Median Sternotomy [EPOCH CardioLink-10]

PI: Ahmad Alli

REB Number: 23-038

Replicate the table below as many times as needed to describe each change.

**Change #1**	Stratification of randomization by site and patient sex
**Rationale:**	Ensure better balance between groups
**Risk:**	None
**Impact:** (e.g. Participant safety, study outcomes, study budget)	No impact on participant safety or study outcomes
**Impacted Documents:**	Protocol

**Change #2**	Increase in sample size from 310 to 314; inclusion of other participating sites in Canada
**Rationale:**	Clarification of sample size calculations; enhance generalizability of study findings
**Risk:**	None
**Impact:**	No impact on participant safety
**Impacted Documents:**	Protocol, ICF

**Change #3**	Removal of neurosurgeons as possible assessors of delirium
**Rationale:**	Neurosurgeons are never involved in assessment of delirium in CVICU patients. This is done by neurologists or psychiatrists (who are still included in protocol).
**Risk:**	None
**Impact:**	None
**Impacted Documents:**	Protocol

**Change #4**	Addition of description of DSMB
**Rationale:**	Clarification of safety monitoring of the study
**Risk:**	None
**Impact:**	Will enhance patient safety monitoring
**Impacted Documents:**	Protocol

**Change #5**	Expansion of description of statistical analyses
**Rationale:**	Clarification of planned analyses including addition of subgroup analyses
**Risk:**	None
**Impact:**	Improved description of analysis of data and outcomes
**Impacted Documents:**	Protocol

**Change #6**	Additional minor changes to text and formatting have been made
**Rationale:**	To improve clarity and consistency of the protocol and ICF
**Risk:**	None
**Impact:**	None
**Impacted Documents:**	Protocol, ICF

Enhanced PeriOperative Cardiovascular Healthcare

EPOCH CardioLink-10 Statistical Analysis Plan (Version 1.0 / 06FEB2026)

STATISTICAL ANALYSIS PLAN

**Protocol Title:**	Efficacy of Superficial Parasternal Intercostal Plane Block on Postoperative Pain Control in Patients Undergoing Cardiac Surgery with Median Sternotomy<br>EPOCH CardioLink-10 Randomized Clinical Trial
**Protocol Number:**	N/A
**Protocol Version/Date:**	30OCT2025
**Investigational Product:**	0.2% ropivacaine
**Sponsor:**	CardioLink Clinical Trial Platform
**SAP Version/Date:**	1.0/06FEB2026

CONFIDENTIAL

The information in this document is confidential and is not to be disclosed without the written consent of the CardioLink Research Platform (CardioLink) except to the extent that disclosure would be required by law and for the purpose of evaluating and/or conducting a clinical study for CardioLink. You are allowed to disclose the contents of this document only to your Institutional Review Board or Independent Ethics Committee and study personnel directly involved with conducting this protocol. Persons to whom the information is disclosed must be informed that the information is confidential and proprietary to CardioLink and that it may not be further disclosed to third parties.

SIGNATURE PAGE

Protocol Title: Efficacy of Superficial Parasternal Intercostal Plane Block on Postoperative Pain Control in Patients Undergoing Cardiac Surgery with Median Sternotomy: EPOCH CardioLink-10 Randomized Clinical Trial

Protocol Number: N/A

SAP Version/Date: 1.0/06FEB2026

We, the undersigned, have reviewed and approved this Statistical Analysis Plan:

Signature **Date**

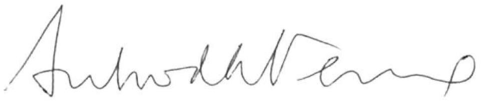

February 6, 2026

Subodh Verma, MD, PhD, FRCSC
Cardiac Surgeon and Professor of Surgery, and Pharmacology and Toxicology
St. Michael's Hospital, University of Toronto

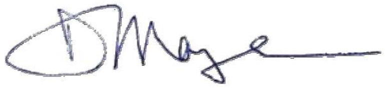

February 6, 2026

C. David Mazer, MD, FRCPC
Anesthesiologist and Professor of Anesthesiology and Pain Medicine, Physiology, and Pharmacology and Toxicology
St. Michael's Hospital, University of Toronto

VERSION HISTORY

Version	Version Date	Description
1.0	06FEB2026	First SAP version

TABLE OF CONTENTS

1 Introduction 7
2 Study Overview 7
2.1 Study Objectives 7
2.1.1 Primary Objective 7
2.1.2 Secondary Objectives 7
2.1.3 Exploratory Objectives 7
2.2 Study Design 7
2.2.1 Overview 7
2.2.2 Sample Size Determination 8
2.3 Study Endpoints 8
2.3.1 Primary Efficacy Endpoints 8
2.3.2 Secondary Efficacy Endpoints 8
2.3.3 Exploratory Efficacy Endpoints 8
2.3.4 Safety Endpoints 9
3 Statistical Methodology 9
3.1 General Considerations 9
3.1.1 Analysis Day 9
3.1.2 Analysis Visits 9
3.1.3 Definition of Baseline 9
3.1.4 Summary Statistics 9
3.1.5 Hypothesis Testing 10
3.1.6 Handling of Dropouts and Missing Data 10
3.1.7 Laboratory Values Above or Below Limits of Quantification 10
3.2 Analysis Populations 10
3.2.1 Modified Intent-to-Treat (mITT) Population 10
3.2.2 Safety Population 11
3.3 Subject Data and Study Conduct 11
3.3.1 Subject Disposition 11
3.3.2 Analysis Populations 11
3.3.3 Demographic and Baseline Characteristics 11
3.3.4 Concomitant Medications 11
3.4 Efficacy Assessment 11
3.4.1 Primary Efficacy Endpoints 11
3.4.2 Secondary Efficacy Endpoints 13
3.4.3 Exploratory Efficacy Endpoints 13
3.4.4 Subgroup Analysis 13
3.5 Safety Assessment 14
3.5.1 Adverse Events (AEs) 14

3.5.2 Postoperative Clinical Laboratory Tests 14
3.5.3 Postoperative Transfusions 14
4 Analysis Timing 14
4.1 Interim Analysis 14
4.2 Final Analysis 14
5 Programming Specifications 14

LIST OF ABBREVIATIONS

Abbreviation	Definition
ANCOVA	Analysis of covariance
BID	Twice daily
CAM	Confusion Assessment Method
CI	Confidence interval
CRF	Case report form
CSR	Clinical study report
HR	Hazard ratio
ICDSC	Intensive Care Delirium Screening Checklist
ICU	Intensive care unit
LOS	Length of stay
LS	Least squares
mITT	Modified Intent-to-Treat
MME	Milligram morphine equivalent
MMRM	Mixed model for repeated measures
OR	Odds ratio
SAP	Statistical analysis plan

1 INTRODUCTION

The purpose of this Statistical Analysis Plan (SAP) is to provide a description of the statistical methods to be implemented for the analysis of data from the EPOCH CardioLink-10 Randomized Clinical Trial. The SAP will be finalized prior to database lock.

2 STUDY OVERVIEW

2.1 Study Objectives

2.1.1 Primary Objective

The primary objective of this study is to evaluate the effect of superficial parasternal intercostal plane (SPIP) blocks with 0.2% ropivacaine on cumulative postoperative opioid use during the 72 hours following catheter insertion into the SPIP.

2.1.2 Secondary Objectives

The secondary objectives of this study include evaluating the effects of superficial parasternal intercostal plane blocks with 0.2% ropivacaine on the following:

- Pain score during the 72 hours following catheter insertion
- Cumulative postoperative opioid use from catheter insertion until discharge from hospital
- Incidence of delirium
- Quality of postoperative recovery

2.1.3 Exploratory Objectives

The exploratory objectives of this study include evaluating the effects of superficial parasternal intercostal plane blocks with 0.2% ropivacaine on the following:

- Time from catheter insertion to extubation
- Time from catheter insertion to first opioid analgesic provision following extubation
- Time from catheter insertion to mobilization
- Intensive care unit (ICU) and hospital length of stay (LOS)
- Incidence of postoperative nausea and vomiting
- Opioid requirements post-discharge
- Chronic post sternotomy pain/disability and patient reported outcomes

2.2 Study Design

2.2.1 Overview

EPOCH CardioLink-10 is a randomized, double-blinded, parallel-group, multicentre study of superficial parasternal intercostal plane blocks with 0.2% ropivacaine versus placebo with 0.9% normal saline administered via catheters in the SPIP placed under ultrasound guidance in patients undergoing cardiac surgery through a median sternotomy. Patients meeting inclusion

criteria who provide informed consent will be randomized in a 1:1 fashion into the intervention or control group. Based on the sample size calculation described in Section 2.2.2, approximately 340 participants will need to be randomized to detect important differences. This will be a multicentre trial at 4 sites in Canada, with usual institutional anesthetic and postoperative analgesia management being provided to all enrolled participants. Participants will be followed prospectively during hospitalization with regular assessments of postoperative pain and recovery, as well as during follow-up contacts at 6 weeks and 3-6 months following their operation.

2.2.2 Sample Size Determination

To detect a mean difference of 10 milligram morphine equivalents (MMEs), assuming a standard deviation of 25 and a 2-sided type 1 error rate of 5%, a sample size of 133 participants per group would be required to provide a power of 90%. Assuming an attrition rate of 25%, approximately 340 patients will need to be randomized.

2.3 Study Endpoints

2.3.1 Primary Efficacy Endpoints

The primary efficacy endpoint is to compare the 0.2% ropivacaine group with the placebo group in terms of cumulative postoperative opioid use measured as MMEs during the 72 hours following catheter insertion.

2.3.2 Secondary Efficacy Endpoints

The secondary efficacy endpoints are to compare the 0.2% ropivacaine group with the placebo group in terms of the following:

- Pain score as measured by a standardized numeric rating scale during the 72 hours following catheter insertion
- Cumulative postoperative opioid use measured as MMEs from catheter insertion until discharge from hospital
- Incidence of delirium during the 72 hours following catheter insertion, as defined by the presence of one of the following criteria: Confusion Assessment Method (CAM) or CAM-ICU positive, or Intensive Care Delirium Screening Checklist (ICDSC) >3, or 3D-CAM, or 4AT ≥4, or more than one dose of haloperidol or similar antipsychotic drug, or documented delirium by neurologist or psychiatrist consultation
- Quality of Recovery-15 Scale administered 24-96 hours post-surgery

2.3.3 Exploratory Efficacy Endpoints

The exploratory efficacy endpoints are to compare the 0.2% ropivacaine group with the placebo group in terms of the following:

- Time from catheter insertion to extubation
- Time from catheter insertion to first opioid analgesic provision following extubation
- Time from catheter insertion to mobilization
- ICU and hospital LOS

- Incidence of postoperative nausea or vomiting, measured via a 10-point visual analogue scale twice daily (BID) and antiemetic medication requirements during the 72 hours following catheter insertion
- Opioid requirements post-discharge as assessed by prescriptions provided at time of discharge and during post-discharge follow-up visit using both patient health record and direct patient recounting
- Chronic post sternotomy pain/disability and patient reported outcomes assessed with the McGill Pain Questionnaire and PROMIS 29 score

2.3.4 Safety Endpoints

The safety endpoints include the following:

- Postoperative laboratory tests
- Postoperative transfusions

3 STATISTICAL METHODOLOGY

3.1 General Considerations

3.1.1 Analysis Day

Analysis day will be calculated from the date of first dose of study treatment. The day of the first dose of study treatment will be Day 0 and the day immediately after Day 0 will be Day 1.

3.1.2 Analysis Visits

Scheduled visits will be assigned to analysis visits as recorded on the case report form (CRF).

For each analysis visit, if a scheduled visit occurs within the analysis day window, then the measurement from this scheduled visit will be used. If no scheduled visit occurs or laboratory results of the scheduled visit were unreportable, the unscheduled measurement closest to the target day will be used. If measurements are equidistant to the target day, the later will be used.

Analysis Visit	Target Analysis Day	Low Analysis Day	High Analysis Day
Day 0 (day of catheter insertion)	0	0	0
Day 42 (phone call visit)	42	28	56
Day 91 (phone call visit)	91	63	119

3.1.3 Definition of Baseline

Baseline is defined as the last measurement prior to the first dose of study treatment, unless otherwise stated.

3.1.4 Summary Statistics

Categorical data will generally be summarized with counts and percentages of subjects. The denominator used for the percentage calculation will be clearly defined. Continuous data will

generally be summarized with descriptive statistics including n (number of non-missing values), mean, median, standard deviation, quartile 1, quartile 3, minimum, and maximum.

3.1.5 Hypothesis Testing

The hypothesis testing of cumulative postoperative opioid use measured as MMEs up to 72 hours following catheter insertion is statistically defined as:

$$H_0: m_1 - m_0 = 0, \; H_1: m_1 - m_0 \neq 0,$$

where m_0 - the mean in the placebo group,

m_1 - the mean in the 0.2% ropivacaine group.

No adjustment for multiple comparisons will be made.

3.1.6 Handling of Dropouts and Missing Data

The objective is for missing data to be kept to a minimum. Continued efforts will be made to measure endpoints on all participants, including those who prematurely discontinued study treatment.

Date Values

In cases of incomplete dates, the missing component(s) will be assumed as the most conservative value possible. For example, if the start date is incomplete, the first day of the month will be imputed for the missing day and January will be imputed for the missing month. If a stop date is incomplete, the last day of the month will be imputed for the missing day and December will be imputed for the missing month. Incomplete start and stop dates will be listed as collected without imputation.

Date imputation will only be used for computational purposes such as treatment-emergent status. Actual date values, as they appear in the original CRFs, will be presented within the data listings.

Non-Date Values

There will be no imputation of missing efficacy or safety data. However, the potential influence of rescue or intraoperative nerve blocks, delayed extubation, return to OR and death on MME efficacy endpoints will be explored in sensitivity analyses, described below.

3.1.7 Laboratory Values Above or Below Limits of Quantification

For laboratory values less than the lower limit of quantification (LLQ), half of the lower limit value (i.e., LLQ/2) will be used in the analysis. For values greater than the upper limit of quantification (ULQ), the upper limit value (i.e., ULQ) will be used in the analyses.

3.2 Analysis Populations

3.2.1 Modified Intent-to-Treat (mITT) Population

The mITT Population will include all randomized participants in whom a block catheter was placed, and a dose of study treatment was administered. Treatment classification will be based on the randomized treatment assignment. The mITT Population will be the primary population used for the efficacy analyses.

3.2.2 Safety Population

The Safety Population will include all participants in whom a block catheter was placed, and a dose of study treatment was administered. Treatment classification will be based on the actual study treatment received. The Safety Population will be the primary population used for safety analyses.

3.3 Subject Data and Study Conduct

3.3.1 Subject Disposition

Counts and percentages of participants who were randomized, completed the treatment period, discontinued treatment (including primary reason for discontinuation), completed the study, and prematurely discontinued from the study (including primary reason for discontinuation) will be summarized by treatment group.

3.3.2 Analysis Populations

Counts of participants in each analysis population will be summarized by treatment group.

3.3.3 Demographic and Baseline Characteristics

The following demographic and baseline characteristics will be summarized with descriptive statistics or counts and percentages of participants as appropriate by treatment group for the mITT Population:

- Site of enrollment
- Sex
- Age
- Race/Ethnicity
- Height
- Weight
- Body Mass Index (BMI)
- History of each of the following: coronary artery disease, valvular heart disease, diabetes, hypertension, dyslipidemia, obesity, smoking within past 6 months
- Medications: as collected in the CRF
- Laboratory assessments: as collected in the CRF
- Preoperative cardiac assessment: as collected in the CRF
- PROMIS-29

3.3.4 Concomitant Medications

Concomitant Medications – other than those included in the efficacy analyses – will be summarized by treatment group for the mITT Population. Intraoperative MMEs will also be summarized.

3.4 Efficacy Assessment

The mITT Population will be the primary population for the efficacy analysis.

3.4.1 Primary Efficacy Endpoints

<u>Primary Analysis</u>

The primary efficacy endpoint is the cumulative postoperative opioid use measured as MMEs during the 72 hours following catheter insertion. The conversion of different opioids into MMEs will utilize the table provided in the study protocol.

Administered opioids are documented in the CRF on a daily basis; i.e., the date and time of each administered drug is not recorded. In contrast, the date and time of catheter insertion is recorded in the CRF. Consequently, opioid use for each participant will include those that were administered:

- The day of catheter administration
- The two days following the day of catheter administration
- A fraction of the third day following the day of catheter administration. The fraction will depend on the time of day that the catheter was inserted at the beginning of the 72 hour time interval. For example, if 40 MMEs were administered on the 3rd day and the catheter was inserted at 9:00 AM on day 0, the MMEs on the third day that will be included in the primary efficacy analysis would be (9/24) × 40 = 15 MMEs.

To assess the primary efficacy endpoint, the primary estimand is defined by the following key attributes:

- **Treatment**: 0.2% ropivacaine versus placebo
- **Target Population**: participants who are randomized into the study and received any amount of study treatment
- **Analysis Population**: The mITT Population
- **Intercurrent events**: premature treatment discontinuation, rescue nerve block, death
- **Analysis set and handling of intercurrent events**: Treatment policy strategy will be used. All available MME values during the 72 hours following catheter insertion will be used in the calculation of cumulative postoperative opioid use
- **Population level summary**: The difference in LS mean cumulative postoperative opioid use during the 72 hours following catheter insertion between treatment groups

The analysis of covariance (ANCOVA) model with a fixed effects for treatment group, sex, BMI and intraoperative MMEs (<median, ≥median) will be used to analyze the primary efficacy endpoint. The least squares (LS) mean, standard errors, and 2-sided 95% confidence intervals (CIs) for each treatment group and for the mean difference compared to placebo will be estimated. The model will be fit assuming unequal variances for each treatment group. If substantial deviations from the model assumptions are observed, then supportive analyses, such robust regression, non-parametric analyses, etc. will be considered. Missing values of predictors will either be imputed by the overall mean or most frequent category.

<u>Sensitivity Analysis</u>

A sensitivity analysis will jointly model the primary efficacy endpoint with incidence of rescue or intraoperative nerve block, delayed extubation, return to the OR or death during the 72 hours following catheter insertion. The primary endpoint will be analyzed using the ANCOVA model described above, and rescue or intraoperative nerve block, delayed extubation, return to the OR

or death occurrence will be analyzed by logistic regression with the fixed effects used in the ANCOVA model.

3.4.2 Secondary Efficacy Endpoints

Pain score during the 72 hours following catheter insertion will be analyzed by a mixed model for repeated measures (MMRM), with random effects for intercept and time, fixed effects for treatment, sex, and intraoperative MMEs (<median, ≥median), and an unstructured covariance matrix.

The analysis of cumulative postoperative opioid use measured as MMEs from catheter insertion until discharge from hospital will be by the same methods as for the primary efficacy endpoint, including the joint model sensitivity analysis.

The analysis of incidence of delirium during the 72 hours following catheter insertion will be by logistic regression with fixed effects for treatment group, sex, and intraoperative MME (<median, ≥median). The treatment odds ratio (OR) and associated 95% confidence interval will be provided.

The analysis of Quality of Recovery-15 Scale administered 24-96 hours post-surgery will be by the same methods as for the primary efficacy endpoint.

3.4.3 Exploratory Efficacy Endpoints

The analyses of time-to-event exploratory efficacy endpoints will be by proportional hazards models with fixed effects for treatment, sex, and intraoperative MMEs (<median, ≥median). The treatment hazard ratio (HR) and associated 95% CI will be provided. A cause-specific approach for competing terminal events (i.e., deaths) will be applied by censoring participants at the time of death. Cumulative incidences by treatment group will be estimated by Kaplan-Meier methods.

The analyses of incidence of postoperative nausea or vomiting will be by the same methods as pain score or delirium.

Opioid requirements post-discharge will be summarized by descriptive statistics.

The analyses of chronic post sternotomy pain/disability and patient reported outcomes assessed with the McGill Pain Questionnaire (3 months) and PROMIS 29 score (6 weeks, 3 months) will be by the same methods as for the primary efficacy endpoint.

3.4.4 Subgroup Analysis

The primary efficacy endpoint will be analyzed by the following subgroups:

- Age (<median, ≥median)
- Race/ethnicity
- Sex (male, female)
- Diabetes status
- BMI (<median, ≥median)
- Type of Operation
- Intraoperative MMEs (<median, ≥median)

An ANCOVA model with a fixed effects for the treatment group, sex, intraoperative MMEs, subgroup (not applicable for sex and intraoperative MMEs subgroup analyses) and the interaction between treatment group and subgroup will be applied. The least squares (LS)

mean, standard errors, and 2-sided 95% confidence intervals for each treatment group and for the mean difference compared to placebo, within each level of the subgroup, will be estimated.

3.5 Safety Assessment

Safety Population will be the primary population for the safety analyses. All safety endpoints will be summarized descriptively by treatment group. No statistical inference will be applied to the safety endpoints.

3.5.1 Adverse Events (AEs)

AEs will be categorized according to the types specified in the CRF and summarized by treatment group.

3.5.2 Postoperative Clinical Laboratory Tests

Postoperative laboratory tests will be summarized with descriptive statistics by treatment group.

3.5.3 Postoperative Transfusions

Postoperative transfusions will be summarized with descriptive statistics by treatment group.

4 ANALYSIS TIMING

4.1 Interim Analysis

No interim analysis is planned.

4.2 Final Analysis

After all comments on the pre-final analysis have been resolved and the study database is declared final, the final analysis will be generated.

5 PROGRAMMING SPECIFICATIONS

Analyses will be performed using SAS® version 9.4 or higher.
